# Functional Data Analysis of high-frequency load curves reveals drivers of residential electricity consumption

**DOI:** 10.1371/journal.pone.0218702

**Published:** 2019-06-25

**Authors:** Matteo Fontana, Massimo Tavoni, Simone Vantini

**Affiliations:** 1 Politecnico di Milano, Department of Management, Economics and Industrial Engineering, Milan, Italy; 2 Politecnico di Milano, MOX - Department of Mathematics, Milan, Italy; 3 RFF-CMCC European Institute on Economics and the Environment, Milan, Italy; University of Maryland College Park, UNITED STATES

## Abstract

Smart energy meters generate real time, high frequency data which can foster demand management and response of consumers and firms, with potential private and social benefits. However, proper statistical techniques are needed to make sense of this large amount of data and translate them into usable recommendations. Here, we apply Functional Data Analysis (FDA), a novel branch of Statistics that analyses functions—to identify drivers of residential electricity load curves. We evaluate a real time feedback intervention which involved about 1000 Italian households for a period of three years. Results of the FDA modelling reveal, for the first time, daytime-indexed patterns of residential electricity consumption which depend on the ownership of specific clusters of electrical appliances and an overall reduction of consumption after the introduction of real time feedback, unrelated to appliance ownership characteristics.

## Introduction

The availability of “Big Data”, i.e. huge and complex datasets, commonly generated by automated data acquisition systems (for example sensor arrays, imaging systems, social networks, search engines, smart meters etc.) is quickly revolutionizing many fields of the mathematical, natural and social sciences. Data scientists have to deal with the increased complexity and size of the data to be processed, developing specific methods, quite different from the ones usually employed in the traditional statistical practice. While other sciences, such as biology [1] or ecology [2] are already employing these very advanced techniques, the immense modelling and interpretative power of Big Data Analytics [3, 4] is being discovered right now by economists, behavioural scientists and policy analysts, including those active in energy and climate change research.

Climate change has shown itself to be one of the biggest and most important policy issues of the 21st century, and an important component of the ‘greening’ of economic systems rests on reducing energy demand at the level of households [5, 6]. Several programmes aimed at increasing energy efficiency in the residential sector have been tested over the years, ranging from economic and informational interventions, to more psychologically oriented programmes [7–15]. The energy industry has moved towards becoming a provider of energy services, enabling individuals to perceive their energy footprint and act on it. In Europe alone almost 200 million smart meters will be installed by 2020, and they will have the ability to provide users with immediate, high frequency feedback on their energy consumption behaviour. Nonetheless, there is mixed evidence about about the effectiveness of smart meter interventions to reduce energy consumption [16–20], and few high quality and high frequency data bases about smart metering exist. While smart meters and consumer demand response techniques can be used as very powerful policy levers, their actual impact has proven quite difficult to assess.

In this paper we analyse an original dataset, coming from a field experiment of real time electricity feedback through in-home displays carried out in Italy during a period of three years. We address two policy-relevant research questions, concerning energy disaggregation and energy saving devices and techniques. Our overarching goal is that of providing an enhanced understanding of consumer behaviour, and to test on the field to what extent high frequency energy data can deliver both energy savings as well as a shift of consumption over the day. This question is paramount since many programmes are now in place not only to incentivize lower energy consumption, but also to promote a more rational use of energy through the day [21, 22]. Currently, typical load curves are characterized by highly variable daily consumption. Being able to smooth energy consumption depending on the available energy sources can provide significant economic benefits, as testified by the significant differences in electricity prices throughout the day, a phenomenon further exacerbated by the increasing penetration of renewable energy sources. High frequency data provides us with temporal consumption patterns which can allow testing for load curve change as a result of having real time feedback. In addition, we assess the drivers of electricity consumption throughout the day, correlating it to household characteristics such as the ownership of electric appliances.

The technique of choice in our case is Functional Data Analysis (FDA) [23–27], a novel branch of Statistics which deals with data that can be represented using a function with some degree of continuity and/or smoothness. The statistical units of FDA are functions, observed in a continuous domain, and lying in a functional space. Thus, FDA is suited at analysing data coming from high frequency readings, such as smart energy meters, or any data with a important temporal dimension. Functional Data Analysis is particularly apt to deal with the intrinsic noisiness of load curves and with their high dimensionality, allowing to detect when during the day a given consumption pattern takes place, and if a reduction in energy consumption is to be attributed to a given appliance or a given set of appliances. Moreover, FDA is able to model not only data curves, but also their derivatives, thus extending the insight one could extract from the data to the rate of change of electricity consumption, an important consideration for the balance of the energy system and the analysis of consumption behaviour. The fitness of FDA techniques in analysing data coming from the energy realm is testified by the presence of some previous works tackling important problems in the energy modelling community, such as the statistical forecasting of demand and supply curves [28–30], the forecasting of household level consumption curves [31], and the analysis of aggregated consumption curves to extract information about households [32]. The objective of our work is different with regards of the previous ones: to our knowledge this is the first attempt to use functional testing techniques in the energy modelling realm, and the first attempt to use functional linear models for energy disaggregation.

## Data

We were granted access to data from a field experiment implemented by a large European energy utility that took place in the years 2012-2014. The project provided households of a province in central Italy with a display able to yield real time feedback on electricity consumption (N = 1064). This In-Home Display (IHD) informs about instantaneous electric power use, as well as daily, weekly and monthly summaries. The device is also able to show the current billing slot, and the time at which the next slot will enter into force. If a customer enters information about billing, the display is also able to provide feedback on monetary expenditures. The device was delivered over a period of almost two years, with the first deliveries in September 2012, while the full scale experiment began a a year later. For a subset of the families involved in the experiment (N = 425), a survey was carried out by the energy utility to gather further data, especially ownership of 34 electrical appliances, as well socio-demographics. We have three different data sources that were merged together: an “Administrative” dataset, where billing and commercial information about energy clients were provided, a “Load Curves” dataset, where high frequency consumption data for given households and for a variable fraction of the 3 years of the experiment was provided and a “Profilation” dataset, that gave us more detailed information about the structure and appliance ownership of the households involved in the experiment.

Electricity load curves are sampled every 15 minutes, meaning that we have 96 measurements for each day in which consumption was measured, for 1064 households.during approximately two years. The Profilation dataset consists of a survey conducted on a subset of 425 household to gather information about the specific households involved in the project, such as the size of the home they live in, and the type of electric appliances they own. A summary of the data gathered can be seen in Table A in S1 File, together with a list of the appliances about which we own ownership data, along with some summary statistics (Table 2 in S1 File). In practice, apart from the social and administrative covariates, we have, for the 425 households involved in the survey, a time series of varying length (across the households) of load curves sampled every 15 minutes.

The combination of high frequency electricity reading with surveys about household characteristics allows us to try answering the following key research questions.

1. Do household electric appliances have a specific “load signature” that can be identified in the various consumption curves?
2. Has the introduction of the smart meter devices affected the energy consumption at the household level, and, if so, does it depend on the type of appliance ownership pattern?

The first question aims at identifying drivers of electricity consumption and its distribution during the day. There are several works in the literature who have looked at the determinants of energy consumption, and some of them include, among other covariates, information about appliance ownership [33, 34]. However -to the best of our knowledge- the existing literature has not looked at the whole temporal domain of daily energy consumption, but rather has focused on aggregated measures such as mean consumption or maximum and idle consumption. Evaluating the load curves for the whole day is an important advancement, given the wide temporal variability of consumption, prices and environmental impacts, and the complexity of consumption patterns. FDA techniques are naturally apt at this kind of investigation. For the second question, the absence of a control group prevents us from carrying out a fully fledged impact evaluation. Nonetheless, comparing differences in consumption before and after having received the IHD at home allows us to test whether there is a specific role for appliances in the change of behaviour which we attribute to having received the smart meter. Moreover to our knowledge, this is the very first attempt in the literature at identifying correlations between appliance ownership and energy consumption reduction.

The main independent variable in our analysis is the ownership of energy technologies, as available from the survey. The stock of energy appliances is an important determinant of electricity use, and most relevant for our analysis, it is likely to influence the temporal distribution of the load curves. We control for other socio-demographics in Section 8 in S1 File. We begin by creating meaningful clusters of similar technologies, and then use FDA techniques to evaluate the temporal impact of technology clusters on electricity consumption throughout the day, its rate of change and the impact of having real time feedback in home displays.

## Clustering of appliances

The problem we are studying, even excluding the response variable that can be handled as a smooth function (and so as a single data point in the *L*^2^ space of doubly continuous functions), is nevertheless inherently high dimensional, since we still have covariates that indicate the presence or absence of a specific appliance in the household (as well as socio-demographics). The complete list of appliances, together with ownership percentages, can be found in Table B in S1 File. Moreover, appliances tend to appear together in households, thus rendering impossible to identify the specific signature of single appliances due to collinearity. To tackle this problem we create groups of appliances that tend to co-occur together, and then test for the signature of those clusters in the electricity consumption data. As an initial step, we carry out a hierarchical clustering procedure [35] on the columns of the dataset, by using a distance appropriate to the problem and the data at hand (see Section 6 S1 File for an extended description and additional details). The procedure has been implemented in the R programming language, using standard functions and the “e1071” package. Observing the dendrogram of the hierarchical clustering procedure performed using Ward Linkage and Hamming distance in Fig C in S1 File, 3 clusters can be clearly identified. These can be interpreted as a group of basic and common appliances (in blue), a group of “high-tech” appliances (in red), and a third one of advanced appliances, that are comparably less technological than the high tech ones, but that are very power intensive (in yellow). In the Section 6 S1 File we also provide a robustness analysis of this clustering procedure.

Based on the hierarchical clustering we now define a set of ownership indexes *I*_*f*,*k*_, ∀*k* *in* {*B*, *H*, *L*}, for each group of appliances identified in the clustering procedure. Let *S*_*B*_, *S*_*H*_, *S*_*L*_ the three groups of appliances, respectively the Basic ones, the advanced Hi-Tech ones, and the advanced Lo-Tech ones, and *N*_*B*_, *N*_*H*_, *N*_*L*_ their respective cluster size. For each household *f*, we define the ownership index of the *k*-th group as:

$$I_{fk}=\frac{1}{N_{k}}\sum_{i=1}^{N_{K}}a_{fki}\forall k\;in\;\left\{B,\;H,\;L\right\}$$ (1)

**Fig 1:**
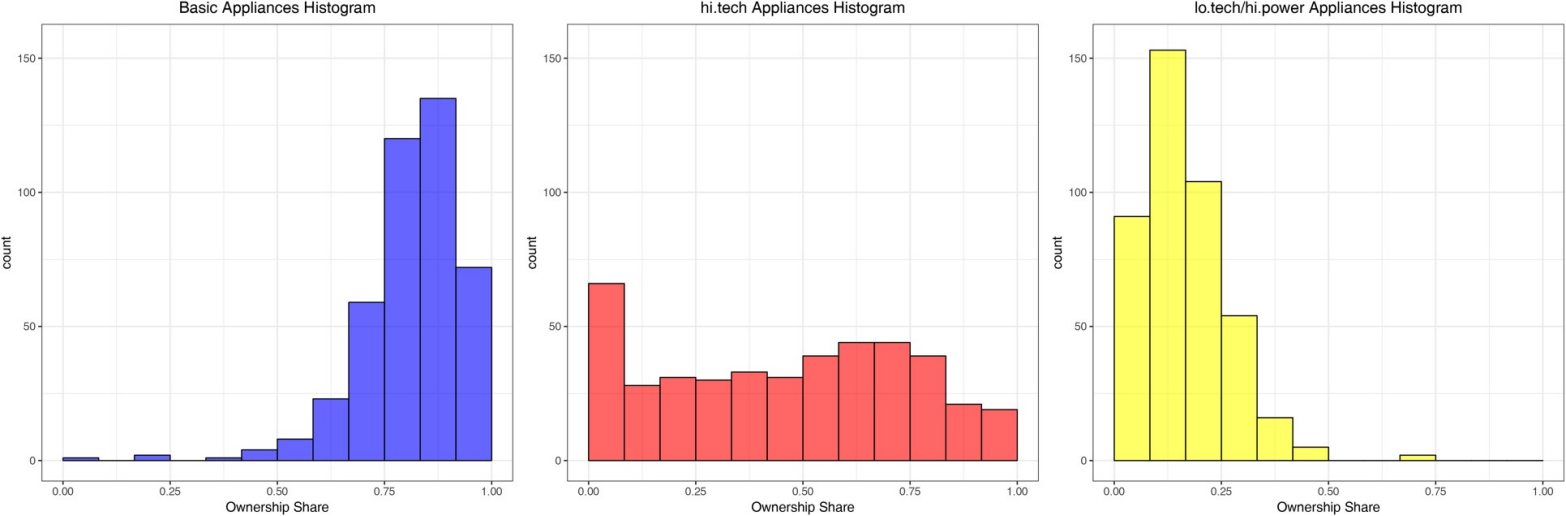
Histograms of the ownership indices of clusters of appliances, generated using the Ward linkage.

Where *a*_*k*,*i*_ is a binary value that indicates the presence or absence of the *i*-th appliance of the *k*-th cluster in the household. In this way we obtain for every household three values, one for each cluster, ranging from 0, meaning that the household does not own any appliance in the given cluster, to 1, which means that the household owns every appliance in the given group. Histograms representing the ownership share of each of the three clusters across households can be seen in Fig 1. The histograms show that while the ownership index of high tech appliances has a somewhat flat distribution, meaning that ownership patterns are widespread among our sample, we observe that both low tech/high power appliances and basic ones have a very specific ownership pattern. People tend to own few lo tech/high power appliances, and many more basic appliances.

## FDA-based modelling and results

The first step performed when dealing with functional data is the extraction of the functional data objects (i.e. the functions that are the objective of the analysis) via a basis expansion procedure. The use of a pre-smoothing technique as reported in [23] is also consistent with the will to focus ourselves on the analysis of the underlying phenomenon, filtering out the measurement noise. However different methods, explicitly modelling the measurement errors, could also be used, such as the one proposed in [36], where the authors employ a mixed-effect model using a B-spline basis, or the one in [37] where the a functional PCA approach is used to reconstruct functional data. Both the calculations and the further regression modelling has been implemented using the R programming language, the “fda” package for smoothing and calculations, and a forthcoming R package for the linear modelling and testing part. The starting point for the construction of our response functions is the extraction of the average discrete load curve for each household *f*, that is:

$$y_{f}=\frac{1}{D_{f}}\sum_{d=1}^{D_{f}}y_{df}$$ (2)

where *y*_*f*_ is the 96 × 1 vector of average consumption values for household *f*, *y*_*df*_ is the vector of consumption values for household *f* in day *d*, and *D*_*f*_ is the number of days for which we have observations of consumption for household *f*. The use of household-specific curves instead of daily ones helps in dealing with the intrinsic measurement noise that such high frequency data have, by averaging it out across days. This approach also solves the problem of eventual missing values.

**Fig 2:**
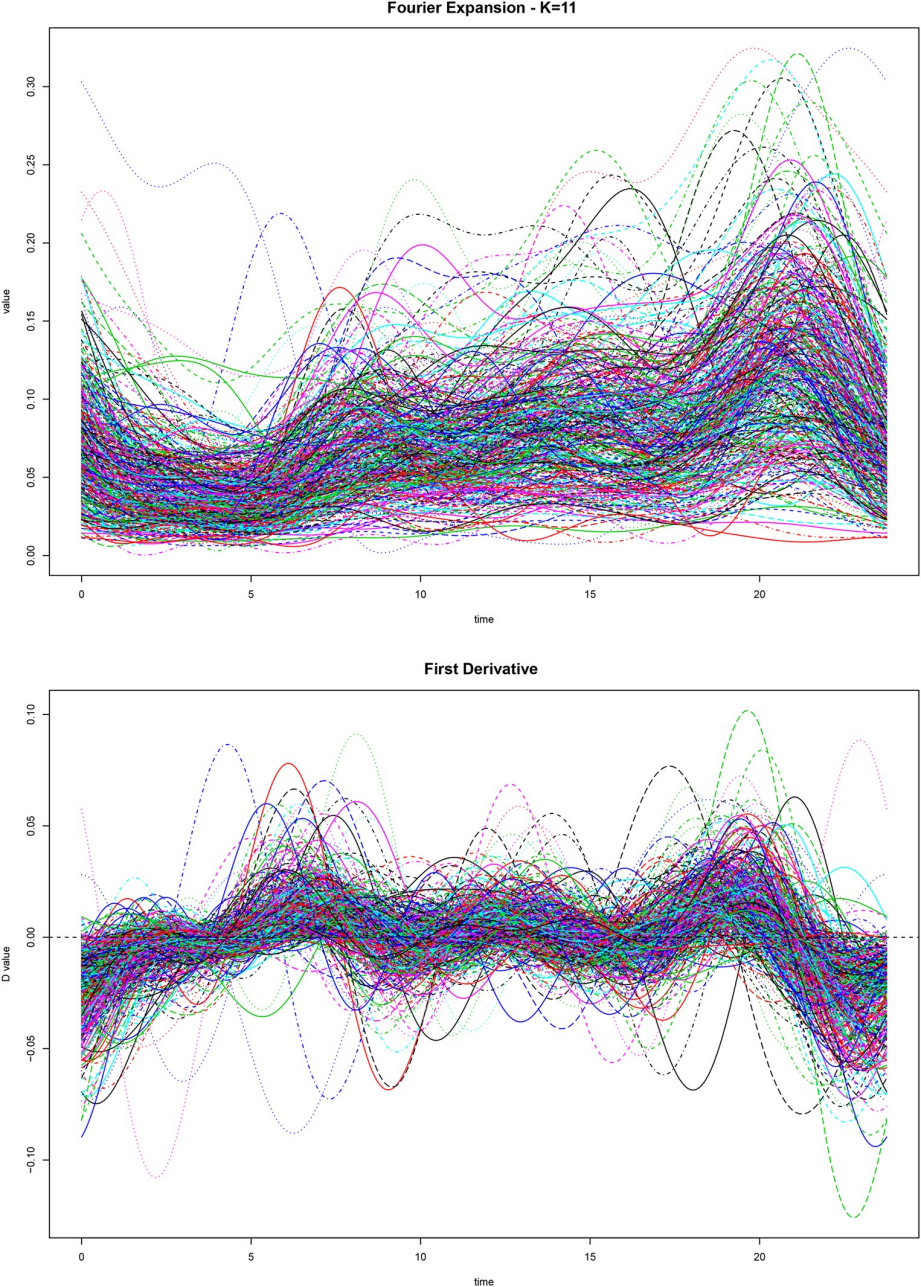
Plot of data smoothed using a Fourier basis of size *K* = 11.

We thus translate the set of discrete 15 minutes electricity meter readings into a continuous function of the time of the day variable *y*(*t*). This operation is performed by using a specific orthogonal functional basis. In this case we used a Fourier basis, due to the periodic nature of the data [23]. As The size of the expansion is performed by looking together at the “wigglyness” of the data curves, and the residual sum of squares. In the case at hand the optimal size of the expansion is *K* = 11, a value that provides a representation of the data that is smooth, but still retains a significant portion of the variability in the dataset. The Residual Sum of Squares (RSS) plot can be seen in Fig D S1 File, while the plot of the smoothed data, together with its first derivative, can be seen in Fig 2.

After the functional smoothing, we can start the FDA analysis by tackling the first research question, namely whether appliance ownership impacts energy use. To assess the impact of the appliances penetration over the consumption curves, we estimate a functional on scalar regression model of appliance ownership on electricity consumption, as follows:

$$y{\left(t\right)}_{f}=\beta_{Baseline}\left(t\right)+\beta_{Hi.tech}\left(t\right)I_{fH}+\beta_{Lo.tech}\left(t\right)I_{fL}+\epsilon {\left(t\right)}_{f}$$ (3)

Where *I*_*f*,*H*_, *I*_*f*,*L*_ are the values of ownership of hi-tech and low tech appliances respectively, *β*(*t*)_*x*_∀*x* *in* {*Baseline*, *ToHi*.*Tech*, *Lo*.*Tech*} are functional regression coefficients, *y*(*t*)_*f*_ is the average energy consumption function over time per household *f* and *ϵ*(*t*) is a zero-mean unpredictable error component, with covariance structure *Σ*(*t*, *t*′). We recall that in this case *n*, the number of statistical units, is 425 households. A preliminary regression analysis, performed for model selection purposes, is presented in Section 7 S1 File. The results of the preliminary model selection regression show that the impact of Basic appliances is not significant, and already captured by the intercept term in a baseline component of energy consumption. Thus, in the remainder of the paper, we will drop the Basic appliances from the regressions. Results for the other groups of appliances are robust to the model specification (see Section 8 S1 File).

**Fig 3:**
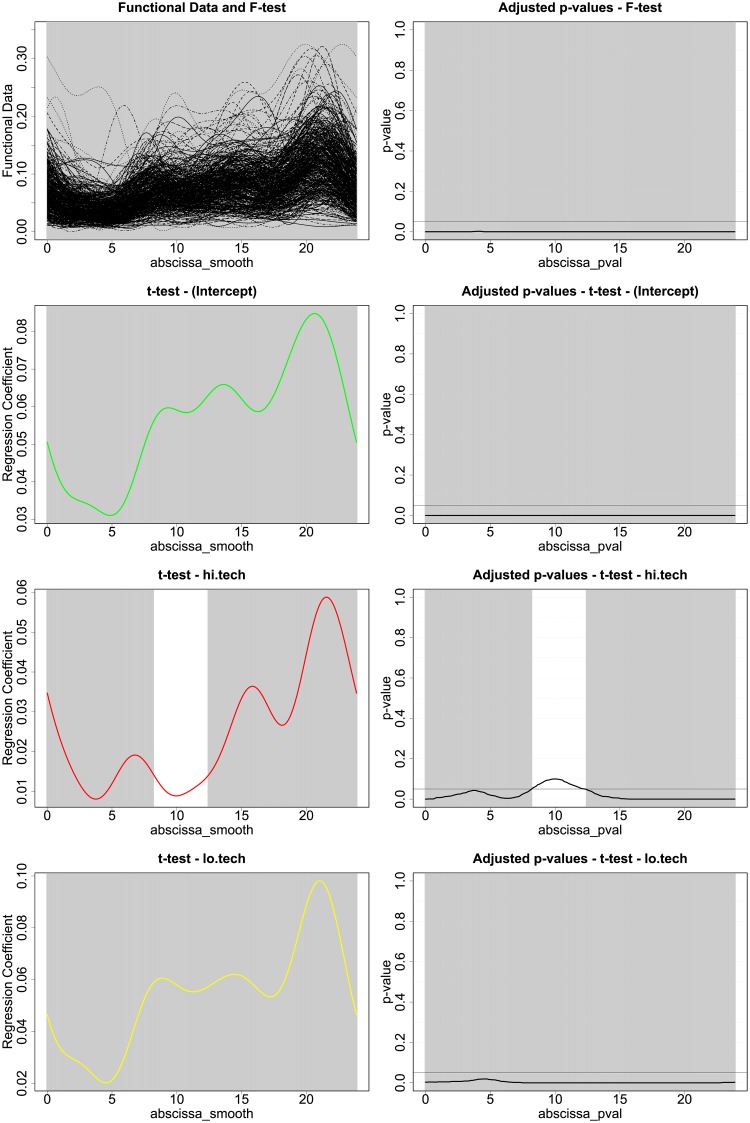
F-Test and t-Tests for regression model in Eq 3.

**Fig 4:**
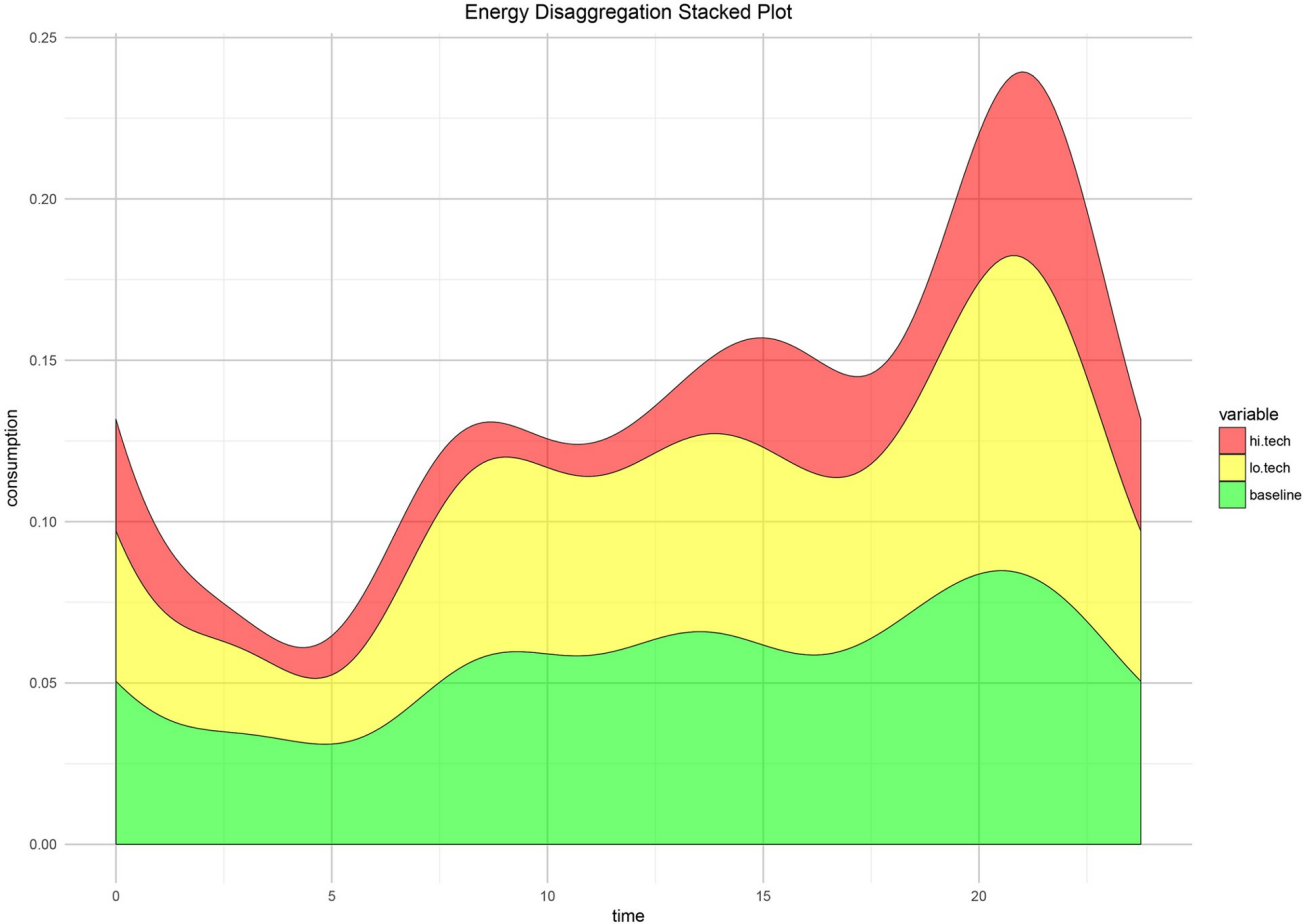
Stacked plot of the coefficients of regression model in Eq 3.

The inferential procedure used to analyse this model are developed in [38], and are based on an interval-wise testing (IWT) framework [39]. A robustness analysis of this inferential procedure with regards to the size of the Fourier basis used for smoothing can be found in Section 8 S1 File. We recall that since our inferential procedures are non-parametric, we neither need to assume a specific probability distribution of the response nor error homoscedasticity across the hours of the day. For all the graphs presented from now onwards, the significance of intervals is represented by a grey shading, meaning that the highlighted area of the domain is significant at global level *α* = 0.05. The results of the tests for the model in Eq 3 can be found in Fig 3. We can see from the F-test conducted over *t* ∈ (0, 24) that the regression is significant over the whole day. We can see from the t-tests and the shapes of the *β*(*t*) that the different terms we used in the regression tell us something specific about energy consumption in a given household. The intercept, shown in green, sets the energy consumption baseline for each household, and its shape resembles very clearly the shape of the mean household consumption curve. An increase in ownership of low tech/high power appliances generates an upward shift of the consumption curve during the whole day. On the other hand, the regression identifies the impact of the ownership pattern of high tech appliances on the energy consumption peaks that can be spotted in the morning, in the afternoon and during the evening. To exemplify this behaviour, we provide a stacked plot of the three functional regression coefficients in Fig 4 (baseline, high tech and low tech).

To deepen our analysis, we test whether owning specific appliance clusters changes not just the quantity of energy consumed, but also the average speed at which energy consumption changes within the day. This is a key question given the importance of balancing in a dynamic way demand and response in the electrical system. We thus model the derivative of the consumption curve, i.e. we write a model of the form:

$$Dy{\left(t\right)}_{f}=\beta_{Baseline}\left(t\right)+\beta_{Hi.tech}\left(t\right)I_{fH}+\beta_{Lo.tech}\left(t\right)I_{fL}+\epsilon {\left(t\right)}_{f}$$ (4)

**Fig 5:**
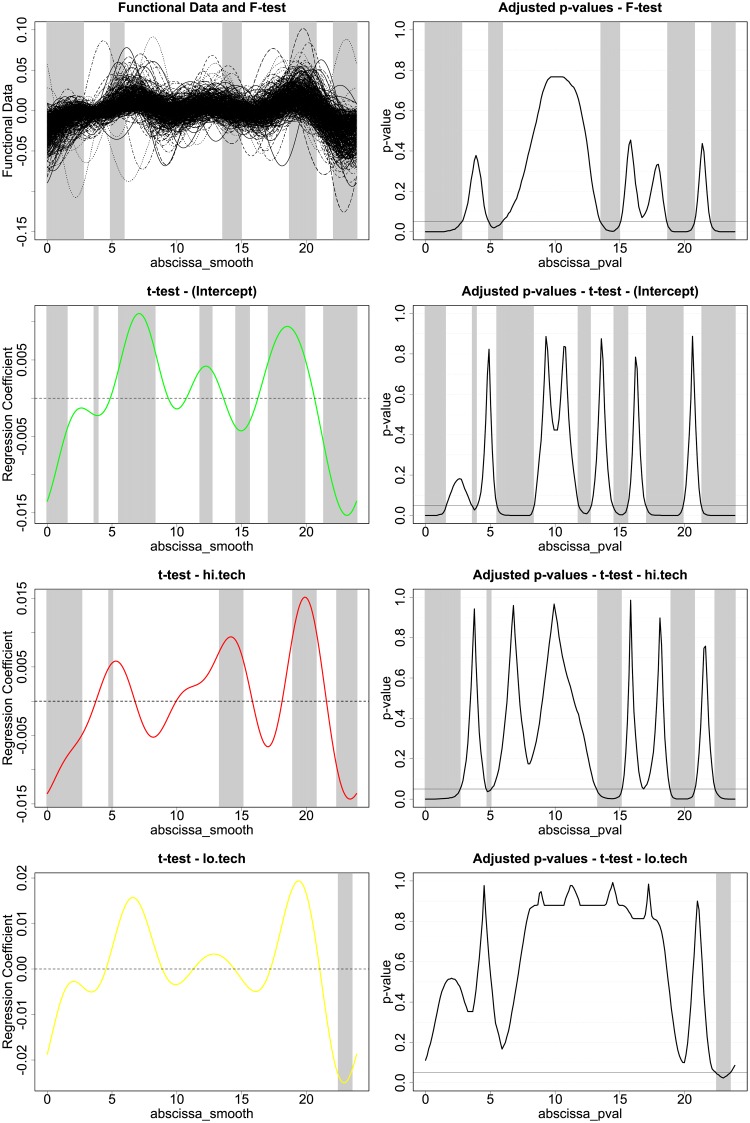
F-Test and t-Tests for regression model 4.

Where *t* ∈ (0, 24), *I*_*B*_, *I*_*H*_, *I*_*L*_ are respectively the values of penetration of basic, hi-tech and low tech appliances. and *Dy*(*t*) is the first derivative of the average energy consumption over time per household and *ϵ*(*t*) is a zero-mean unpredictable error component, with covariance structure *Σ*(*t*, *t*′). From the results in Fig 5 we notice that the value of *I*_*H*_ does influence the derivative of the consumption curve, that can be interpreted as the speed of change of the electric consumption, confirming the magnitude shift relative to the second cluster of appliances. In particular, we notice a sudden increase in the energy consumption in the early afternoon and at dinner time (20:00), followed by a re-stabilization at approximately 16:00.

To test the robustness of our disaggregation model, we have varied the specification of the functional regression equation by adding additional household-specific covariates. In particular, we have inserted the number of people living in a given household, the number of rooms the house is composed, and a dummy variable that is 0 if the house is a flat or an apartment, and 1 otherwise (independent houses, villas etc.). The results show that, for the original covariates (the ownership indices) the portions of the domain that are significant are slightly smaller than in the original regressions, and the magnitude and shape of the coefficients remain the same. The baseline component, instead, stops to be significant after the introduction of additional covariates. This fact is explained by thinking about what the Baseline component was modelling in the previous regression: The role of the baseline was to explain all those energy consumption components that are not appliance ownership (among all, electric lighting and energy dispersions). When you insert more direct measures of these household specific features, those become significant instead of the simple, and less explicative, Baseline.

We keep the same robustness specification for the analysis of the functional regression model for the derivative. The results are very similar to the previous case: some significance is lost for the ownership indexes coefficients, while magnitude and shape is preserved. The Baseline component ceases to be significant, and is replaced by the household specific covariates inserted, that are a better representation of the non-appliance related consumption.

We now turn to our second research question, namely whether appliance ownership matters for changing energy consumption habits after having received the smart meter device. To verify this hypothesis, we employ a Functional difference in differences approach writing a functional on scalar regression model where the response function is the logarithmic differences between an “after” consumption pattern, and a “before” one, to focus on percentual variations of consumption influenced by percentual variations of appliance ownership.

Let *D*_*x*_ be the day when the smart information device has been delivered to a specific household, we define

$$y{\left(t\right)}_{f\;Before}=\frac{1}{N_{1}}\sum_{d=1}^{D_{x}}y{\left(t\right)}_{df}$$ (5)

Where *N*_1_ is the number of days in the interval (1, *D*_*x*_), and

$$y{\left(t\right)}_{f\;After}=\frac{1}{N_{2}}\sum_{d=D_{x}+20}^{D_{x}+20+N_{2}}y{\left(t\right)}_{df}$$ (6)

Where *N*_2_ is the number of days that we have from 20 days after the delivery date to the end of the measuring period. It is reasonable to assume, that the actual variation in the consumption patterns can be seen as percentage of the original value, so we compute the log-differences in the following way:

$$log\Delta {\left(t\right)}_{f}=\text{log}(\frac{y{\left(t\right)}_{f,After}}{y{\left(t\right)}_{f,Before}})=\text{log}\left(y{\left(t\right)}_{f,After}\right)-\text{log}\left(y{\left(t\right)}_{f,Before}\right)$$ (7)

The regression model we specify is the following, a log-log functional-on-scalar regression model of the form:

$$log\Delta {\left(t\right)}_{f}=\beta_{Intercept}\left(t\right)+\beta_{Hi.tech}\left(t\right)\text{log}\left(I_{f,H}\right)+\beta_{Lo.tech}\text{log}\left(I_{f,L}\right)+\epsilon \left(t\right)$$ (8)

**Fig 6:**
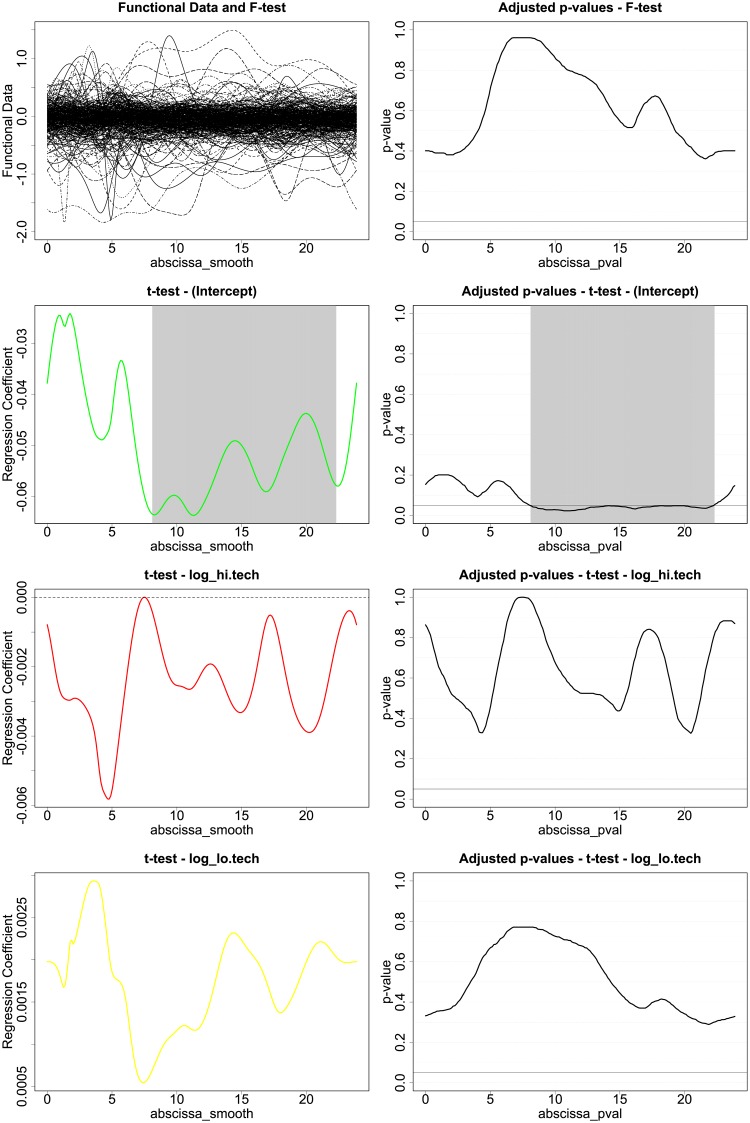
F-Test and t-Tests for regression model in Eq 8.

By looking at the tests on the functional diff-in-diff regression in Fig 6. we can see that the tests are not significant, meaning that the specified covariates do not explain the differences in consumption induced by the in home real time feedback display. If we look at the t-tests, we notice that the coefficient functions *β*_*Hi*.*Tech*_(*t*), *β*_*Lo*.*Tech*_(*t*) are not significant across the whole domain *t* ∈ (0, 24), while the intercept is significant during the day and in the evenings. This means that a general decrease in energy consumption during specific part of the day can be identified, and quantified by looking at the *β*_*intercept*_(*t*) in almost 0.06 = 6%. Nonetheless, given the absence of significance of the coefficient functions multiplying appliance clusters ownership indexes, the decrease in consumption appears not to be influenced by the electrical appliances a household owns.

We have performed the same robustness checks as before also on the difference in differences model, by adding the logs of the same set of household specific covariates. In this case, the intercept coefficient becomes non-significant, and non-significant are also the household specific covariates. This means that there is correlation between the regressors, but that indeed some part of the energy savings can be attributed to specific household components. However, the signal is too weak to appear statistically significant.

## Policy conclusions and limitations

In this work we tackled a policy relevant problem in the realm of energy economics and energy analytics using a novel technique, namely Functional Data Analysis (FDA). FDA has that helped identifying drivers of electricity consumption, its derivative and the impacts of having received a real time in-home display on the whole daily, temporal load. Results have revealed time varying patterns of energy consumption and their relation with ownership of three different clusters of electric appliances. Depending on the time of day, ownership of appliances belonging to different clusters drives electricity consumption and its instantaneous change. We also use the fact that the households in our sample have received a display providing real time information on their consumption to determine whether appliance ownership has determined a change in daily use of electricity. Comparing pre- and post- intervention consumption, the analysis indicates that despite we observe a statistically significant drop in electricity use, this doesn’t appear to be related with the structure of ownership of appliances. This is an important and novel insight, given the policy request to understand who should be the to target of these behavioural and informational interventions [40] and the absence of this kind of analysis in the current literature [41]. Previous research has documented significant heterogeneity in aggregated energy demand response to energy efficiency programs [42–44], with socio-demographics and historical consumption showing up as important mediators. Despite our sample showing a large distribution of energy consumption as well as of ownership of appliances, the statistical results point to a limited role of energy-using technology on the effectiveness of providing residential users with real time information about energy use and costs. The context of a regulated market with limited price differentiation between times of the day might help explain this absent correlation. Further studies carried out with state of the art randomized controlled trials should help investigate these issues further. Only by deepening our understanding of the levers which can enable smart technologies to work, we can fully evaluate their cost effectiveness and the case for specific policy support.

## Supporting information

S1 File Table A, Summary of the data gathered in the context of “Progetto Isernia”. Table B, List of Appliances with contingency table and ownership percentages. Table C, Cophenetic coefficients of the various linkage techniques used. Fig A, Graphical representation of the Hamming distance matrix. Fig B, Dendrograms generated using different linkage methods. Fig C, Ward linkage dendrogram with highlighted clusters. Fig D, Residual sum of squares of fourier expansion. Fig E, F-Tests and t-Tests for regression model in Equation 21. Fig F, F-Test and t-Tests for regression model in Equation 22, *K* = 7. Fig G, F-Test and t-Tests for regression model in Equation 22, *K* = 23. Fig H, F-Test and t-Tests for regression model 23. Fig I, F-Test and t-Tests for regression model 24. Fig J, F-Test and t-Tests for regression model 25.
(PDF)
