# Supplementary material for: Functional Data Analysis of high-frequency load curves reveals drivers of residential electricity consumption
Source: PLoS One. 2019 Jun 25;14(6):e0218702. doi: 10.1371/journal.pone.0218702 (PMC6592564; doi:10.1371/journal.pone.0218702)
Supplement: S1 File — Table A, Summary of the data gathered in the context of “Progetto Isernia”. Table B, List of Appliances with contingency table and ownership percentages. Table C, Cophenetic coefficients of the various linkage techniques used. Fig A, Graphical representation of the Hamming distance matrix. Fig B, Dendrograms generated using different linkage methods. Fig C, Ward linkage dendrogram with highlighted clusters. Fig D, Residual sum of squares of fourier expansion. Fig E, F-Tests and t-Tests for regression model in Equation 21. Fig F, F-Test and t-Tests for regression model in Equation 22, K = 7. Fig G, F-Test and t-Tests for regression model in Equation 22, K = 23. Fig H, F-Test and t-Tests for regression model 23. Fig I, F-Test and t-Tests for regression model 24. Fig J, F-Test and t-Tests for regression model 25. (PDF) [file pone.0218702.s001.pdf]

Supplementary Material

0.1 Functional Data Analysis

Functional Data Analysis, or FDA [3,22,24] is the branch of Statistics that deals with the theory and analysis of data that come come in form of functions, shapes, images, or even more exotic objects such as trees [26].
While the term «Functional Data Analysis» was coined by [21]and [23], the history of this area is much older, and can be traced back to the pioneering works of [7] and [25]
In a more precise way, the data objects that are handled by FDA methods are one or more functions, observed in a continuous domain, and lying in a functional space. The most common choice is the Hilbert separable L^2 space of square-integrable functions, there are nevertheless several generalizations, such as the use of metric spaces by [4,5].

The FDA framework is particularly useful to deal with those cases, increasingly common in the applications, given the pervasivity of data-acquisition devices, where p, the number of features recorded for each sample unit is bigger, or comparable in size with the number n of sample units themselves.
The proper term used to describe this situation is *Large p - Small n.*

Given the functional nature of the data objects analyzed, $p = \infty$, while n is clearly $< \infty$.
In the common practice, this kind of data objects are projected on a finite dimensional space, spanned by a suitably truncated functional basis, that can be fixed or data driven in some way. The most common choices are Fourier basis for periodic data, wavelets for spatially located data, Polynomial basis, B-splines, or basis defined by the Karhunen–Loève decomposition of the autocovariance operator, the so called Functional Principal Components (FPCA).

While several traditionally multivariate techniques (such as regression, k-means and hieararcal clustering, classification and principal component analysis) have already been ported to the functional realm (a good state of the art summary can be found in [29]) inference is still an open and challenging topic in FDA.
We start to describe the extension of the linear regression to the functional realm, then we will see how inference is a problematic issue within FDA, and how specific problems that arise with functional data are solved.

0.2 Functional Regression

The Functional-on-Scalar Linear Model.

Suppose we have observed a set of n continuous square-integrable random functions denoted by $y_i(t)$ over a domain $T = [a,\ b] \subset \mathbb{R}$ where $t \in T,\ i = 1, ..., n$,and $y_i(t) \in L^2[a,\ b]\ \cap\ C^0[a,\ b],\ \forall i \in \mathbb{R}$.

We want to analyze a functional on scalar linear model of the form:

$$y_i(t) = \beta_0(t) + \sum_{l=1}^{L} \beta_l(t) x_{li} + \epsilon_i(t); \qquad i = 1, ..., n \; t \in [a, b] \tag{1}$$

where $x_{1i}, ..., x_{Li} \in \mathbb{R}$ are known scalar covariates and $\beta_l(t),\ l = 0, ..., L$ are the unknown fixed functional regression parameters. We assume the errors $\epsilon_i(t)$ to be i.i.d. zero-mean random functions (we do not need them to be normal), with finite total variance, that is:

$$\int_a^b \mathbb{E}[\epsilon_i(t)]^2 dt < \infty, \qquad i = 1, ..., n \tag{2}$$

Model Estimation

The ordinary least squares (OLS) estimates of the functional parameters β_l, can be found by minimizing, like it is usually done in multivariate statistics [11], the functional equivalent of the residual sum of squares (RSS).
In this case, it is the sum over units of the squared L^2distances between the functional data $y_i(t)$ and the predicted value $\beta_0(t) + \sum_{l=1}^{L} \beta_l(t) x_{li}$with respect to β_l [13, 24], hence minimizing the following quantity:

$$\sum_{i=1}^{n} \int_T \left(y_i(t) - (\beta_0(t) + \sum_{l=1}^{L} \beta_l(t) x_{li} \right)^2 dt \tag{3}$$

For the interchangeability of summation and integration in the former equation, we can say that the minimization can be done separately for each point of the domain, independentely from the covariance structure of the errors $\epsilon_i(t)$.
The OLS estimates $\hat{\boldsymbol{\beta}}(t) = [\hat{\beta_0}(t), ..., \hat{\beta_L}(t)]',\ t \in T$ can so be calculated pointwise for each $t \in T$ as:

$$\hat{\boldsymbol{\beta}}(t) = argmin_{\beta_0(t),...,\beta_L(t)} \sum_{i=1}^{n} \left(y_i(t) - (\beta_0(t) + \sum_{l=1}^{L} \beta_l(t) x_{li} \right)^2 \tag{4}$$

We can see that for each $t \in T$, $\hat{\boldsymbol{\beta}}(t)$ is the OLS estimator of the corresponding scalar on scalar multivariate linear model at point t.
The asymptotic properties for the OLS estimates can be directly derived from the classical results for scalar on scalar linear models. Let $\mathbf{X}$ be the design matrix, $\mathbf{X}_n \in \mathbb{R}^{(n \times (L+1))}$, if the following conditions hold

1. The matrix $\mathbf{X^T X}$ is non-singular, and therefore $\mathbf{V} = (\mathbf{X^T X})^{-1}$exists

2. For each $t \in T$, the regression errors $\epsilon_i(t)$ satisfy:

$$var(\epsilon_i(t)) < \infty$$

we can say that for each t the OLS estimate

$$\hat{\boldsymbol{\beta}}(t) = (\mathbf{X}^{\mathbf{T}}\mathbf{X})^{-1}\mathbf{X}^{\mathbf{T}}\boldsymbol{y}(t) \tag{5}$$

Is a strongly consistent estimate of $\boldsymbol{\beta}(t)$ [16].
Condition 1 is a sufficient condition to find an explicit expression of the OLS estimates, and guarantees convergence in probability, Condition 2 ensures almost sure convergence.

0.3 Inferential Tools for FDA

The classical multivariate inferential tools, such as the Hotelling theorem, require the number of statistical units to be larger than the dimension of the space in which the data are observed, an assumption that is clearly not satisified for functional data, that is naturally infinite-dimensional.
For this reason, several attempts at performing inference for these kind of data objects were attempted: in particular, methods have been proposed for the Functional Analysis of Variance (FANOVA) problem in [31] and under parametric assumptions in [12].

In the case of FDA, the standard parametric assumptions (gaussianity, above all others) are optimistic at best. Moreover, testing the normality of a dataset in a functional framework is impossible, given the fact that the projection of the data over every element of the functional space should be proven to be normal- the normality assumption is not veriable in the Functional Data Analysis framework. In fact, it implies that the projections of the functional data over every element of the functional space are normally distributed [27].
The most rational solution to this problem is to resort to nonparametric methods, usually based on permutation testing tecniques [2] or on the bootstrap [8, 9].

Another critical problem in the tecniques used for inference in the FDA framework is that almost every one of them, both nonparametric or parametric, are global tests providing a unique result based on the weak control of the *Family Wise Error Rate (FWER)* over the domain, i.e. the control of the global level of the test.
In a nutshell, these methods tell you if there is enough evidence to reject the null hypothesis, without being able to tell which parts of the domain are responsible for such rejection.

The methodologies proposed in [19](Inteval Testing Procedure, ITP) and in [18] (Interval-Wise Testing, IWT) are designed to provide the portions of the domain that are actually responsible for the rejection of the null hypothesis, and so influence in a significant way the outcome variable, without sacrificing too much statistical

power. [18] considers [19, 28] as a starting point, exploiting the properties of both approaches.
In detail, it suggests a testing procedure that does not rely on some kind of basis expansion for data or a priori domain partition in sub-intervals, to obtain a fully non-parametric inferential procedure able to detect those parts of the domain responsible for the rejection of the global nulll hypothesis H_0, for functional data embeeded in L_2.

Let us suppose that, after observing a set of random functions embeeded in L^2, over a generic domain $D = (a,\, b) \subset \mathbb{R}$, we want to test a null hypothesis H_0 about our functional data, against an alternative one H_1. For simplicity, we consider a two sample situation, where $\mu_1$and $\mu_2$are the means of the two functional population at hand and $\mu_1,\, \mu_2 \in L^2(T)$. We can write the null hypothesis H_0 as $\mu_1 = \mu_2$, and the alternative one as $\mu_1 \neq \mu_2$.
Let be$\mathcal{I} \subseteq D$ an interval or a complementary interval of the form $\mathcal{I} = (t_1,\, t_2)$,or $\mathcal{I} = X \setminus (t_1,\, t_2)$, where $a \leq t_1 \leq t_2 \leq b$.
We can also define as $H_0^{\mathcal{I}}$and $H_1^{\mathcal{I}}$the restriction of the null and the alternative hypotheses on $\mathcal{I}$.
If we consider as before a two sample situation, we have $H_0^{\mathcal{I}} \,:\, \mu_1^{\mathcal{I}} = \mu_2^{\mathcal{I}}$, and $H_1^{\mathcal{I}} : \mu_1^{\mathcal{I}} \neq \mu_2^{\mathcal{I}}$, where $\mu_1^{\mathcal{I}},\, \mu_2^{\mathcal{I}}$are the restrictions of μover $\mathcal{I}$.

In the most general case, given an $\mathbb{R}$-valued functional test statistic that could be used to test in a global way H_0, the suggested procedure is composed of these sequential steps:

1. Interval-wise Testing: for any $\mathcal{I} \subseteq T$, $p^{\mathcal{I}}$is defined as the p-value of the functional test of $H_0^{\mathcal{I}}$versus $H_1^{\mathcal{I}}$, taking into account the restriction of the chosen test statistic over $\mathcal{I}$.

2. Definition of the p-value functions: the unadjusted and adjusted p-value functions, respectively $p(t)$ and $\tilde{p}(t)$ are defined as:

$$p(t) = \lim_{\mathcal{I} \to t} p^{\mathcal{I}}(t); \qquad \tilde{p}(t) = p^{\mathcal{I}}(t),\ \mathcal{I} \ni t$$

   where with $\mathcal{I} \to t$ we mean that both the extremes of $\mathcal{I}$ converge to t.

3. Domain Selection: the intervals where the test is significant in a point-wise or interval-wise sense are obtained by applying a specified threshold α respectively to the unadjusted p-value function $p(t)$ or to the adjusted one $p^{\mathcal{I}}(t)$.

Linear Model Inference by Interval-Wise Testing

We now describe further how the IWT can be used to perform inference over a Functional-on-Scalar linear model. Clearly, everything described here can be gener-

alized and extended to more complex inferential problems.
In a regression framework, we are usually interested in two kind of tests:

- Overall testing - i.e. a functional version of the F-Test of the classic multivariate regression, that can be written in this way:

$$\begin{cases} H_{0,F} : \beta_l(t) = 0 \quad \forall l \in 1, ..., L, \ \forall t \in D \\ H_{1,F} : \beta_l(t) \neq 0 \quad for\ some\ l \in 1, ..., L,\ and\ some\ t \in D \end{cases} \tag{6}$$

- Tests over the single functional parameters - i.e a functional version of the t-test:

$$\begin{cases} H_{0,l} : \beta_l(t) = 0 \quad \forall t \in D \\ H_{1,l} : \beta_l(t) \neq 0 \quad for\ some\ t \in D \end{cases} \tag{7}$$

We can actually think about this inferential problems as particular cases of the general linear functional hypothesis testing, specified by a combination matrix $\mathbb{C} \in \mathbb{R}^{(q \times (L+1))}$,where q is the number of linear hypotheses over the functional parameters to be jointly tested.
Let also be $\mathbf{c_0}(t) = (c_{0,1}(t), ..., \ c_{0,q}(t))^T$be a vector of fixed functions in $L^2[a,\ b] \cap C^0[a,\ b]$. The general testing problem can so be formulated in these terms:

$$\begin{cases} H_{0,\mathbb{C}} : \mathbb{C}\boldsymbol{\beta}(t) = \mathbf{c}_0(t) \quad \forall t \in D \\ H_{1,\mathbb{C}} : \mathbb{C}\boldsymbol{\beta}(t) \neq \mathbf{c}_0(t) \quad for\ some\ t \in D \end{cases} \tag{8}$$

Where the j-th element of the vector $\mathbb{C}\boldsymbol{\beta}(t)$ is a function obtained by the linear combination of the functional regression parameters.
In this way, we can write the hypothesis in Equation 6 if we let $q = L,\ \mathbb{C} = \mathbb{C}_F = (0|\ I_L) \in \mathbb{R}^{L \times (L+1)}$and $\mathbf{c}_0(t) = \mathbf{0} \in \mathbb{R}^L$,where $I_L$is an identity square matrix of size L.
We can also write the hypothesis in Equation 7 if let $q = 1,\ \mathbb{C} = \mathbb{C}_L = \in \mathbb{R}^{1 \times (L+1)}$with $[\mathbb{C}_l]_r = 1$ if $r = l$ and 0 otherwise, and $c(t) = 0$.

In the case of rejection of $H_{0,\mathbb{C}}$, we would like to identify and select those intervals in T that are responsible for the rejection of the global functional hypothesis. In theory, this problem can be solved by performing an infinite family of tests along T, of the form:

$$\begin{cases} H^t_{0,\mathbb{C}} : \mathbb{C}\boldsymbol{\beta}(t) = \mathbf{c}_0(t) \\ H^t_{1,\mathbb{C}} : \mathbb{C}\boldsymbol{\beta}(t) \neq \mathbf{c}_0(t) \end{cases} \tag{9}$$

Essentially, we are performing an uncountable infinite number of dependent hypothesis tests, that, while they may be easy to implement (since they are straightforward

scalar on scalar tests), they present a considerable challenge in terms of familywise error rate.

We can extend the IWT procedure presented in [18], to functional on scalar linear models. The three steps of the domain selection procedure assume this form:

1. *Interval-Wise Testing*: Given any closed interval $\mathcal{I} \subseteq T$, we want to test:

$$\begin{cases} H_{0,\mathbb{C}} : \mathbb{C}\boldsymbol{\beta}(t) = \mathbf{c}_0(t) & \forall t \in D \\ H_{1,\mathbb{C}} : \mathbb{C}\boldsymbol{\beta}(t) \neq \mathbf{c}_0(t) & for\ some\ t \in D \end{cases} \tag{10}$$

The test statistic we decide to use is:

$$T_{\mathbb{C}}^{\mathcal{I}} = \int_{\mathcal{I}} T_{\mathbb{C}(t)} dt \tag{11}$$

where

$$T_{\mathbb{C}}(t) = (\mathbb{C}\hat{\boldsymbol{\beta}}(t) - \mathbf{c_o}(t))^T(\mathbb{C}\hat{\boldsymbol{\beta}}(t) - \mathbf{c_o}(t)) \tag{12}$$

and $\hat{\beta}(t)$ is the OLS estimate. In particular, for the overall model hypothesis on $\mathcal{I}$

$$\begin{cases} H_{0,F}^{\mathcal{I}} : \beta_l(t) = 0 & \forall l \in 1, ..., L,\ \forall t \in \mathcal{I} \\ H_{1,F}^{\mathcal{I}} : \beta_l(t) \neq 0 & for\ some\ l \in 1, ..., L,\ and\ some\ t \in \mathcal{I} \end{cases} \tag{13}$$

we use the following test statistic

$$T_F^{\mathcal{I}} = \int_{\mathcal{I}} \sum_{l=1}^{L} \hat{\beta}_l(t) dt \tag{14}$$

Instead, we define the hypotheses on the lth regression parameter over $\mathcal{I}$

$$\begin{cases} H_{0,l}^{\mathcal{I}} : \beta_l(t) = 0 & \forall t \in \mathcal{I} \\ H_{1,l}^{\mathcal{I}} : \beta_l(t) \neq 0 & for\ some\ t \in \mathcal{I} \end{cases} \tag{15}$$

and the test statistic is

$$T_F^{\mathcal{I}} = \int_{\mathcal{I}} \left(\hat{\beta}_l(t)\right)^2 dt \tag{16}$$

2. *Adjustment of p-value functions:* The permutation scheme used to calculate the tests over the functional parameters of the model is based on Freedman-Lane permutation scheme [6] as described by [17].

Data Family	Data Type
Administrative Data	Client ID
	In House Display (IHD) delivery date
	Municipality
	Display version
	Presence of microgeneration devices
	Contractual power
Profilation Data	Number of people in the household
	Age and sex
	Electric Appliances owned (34 items)
	Size of the household (number of rooms)
Load Curves	Sampled every 15 minutes

Table A: Summary of the data gathered in the context of «Progetto Isernia»

This particular approach is the most commonly used in the linear modelling framework, since, it can be shown empirically that its statistical power is normally higher than the power of tests based on other permutation schemes [1, 30].Following the idea that guided us during this part, we can define the adjusted p-value functions for the F-test and the individual parameter testing as

$$\tilde{p}_F(t) = \sup_{\mathcal{I}\ni t} p_F^{\mathcal{I}}; \quad \tilde{p}_l(t) = \sup_{\mathcal{I}\ni t} p_l^{\mathcal{I}}; \qquad t \in D$$

3. *Domain selection:* the parts of the domain where the null hypothesis can be rejected are identified by setting a specific threshold α on the adjusted p-value function.

0.4 Data Source, Privacy and Availability

All the data used in the paper were provided after the signing of a Non-Disclosure agreement between the research group and the utility company. The data recieved by the research group were previously fully anonymized, in accordance to the European laws about privacy.

All data needed to replicate the analyses presented in the paper are publicly available as .rdata archive on the Center for Open Science data repository, osf.io, at the following link: https://osf.io/42wb8

0.5 Data Table

We present in Table A a summary of the data available in the Administrative, Profilation and Load Curves dataset

Moreover, in Table B, we present the list of appliances, with ownership percentages

0.6 Dimensionality Reduction of Appliance Covariates via Clustering and Robustness Checks

Introduction

One of the first problems we have encountered is the considerable dimensionality of the energy disaggregation and demand response analysis problems.
Apart from the response variable, that will be treated as a continuous function and not as vector of 96 variables, with some kind of correlation structure between them, we still have the 34 covariates that indicate the presence or absence of a specific appliance in the household.
Moreover, it would be interesting to find if some homogeneous groups can be found in the data, and to find out if such groups show peculiar features in the energy consumption patterns.

A very natural choice for these kind of tasks is to use methods based on Principal Component Analysis (PCA) [14]. Unfortunately, given the binary nature of the appliance presence data, the idea of using a technique explicitly developed to tackle gaussian data is not the most wise one, since we are not able to define in a meaningful way a correlation matrix for binary data, and thus we lack the very item that is required to perform PCA.

The idea is instead to create groups of appliances by using a clustering technique, specifically a hierarchical agglomerative clustering method, as described in [11].

Appliances Clustering

Choice of the appropriate distance

The starting point of every clustering method is the representation of data in terms of proximity between pairs of data objects. This representation can be in terms of similiarities or dissimililarities (respectively alikeness or affinity and difference or lack of affinity).
The idea is to create a $N \times N$ matrix $\mathbf{D}$, where $d_{i,j}$ represents the distance between the i-th and the j-th element.
We have chosen to use the so called *Hamming distance* [10], since it is the most natural and data-driven way to handle binary vectors such as the columns of household-level incidence of appliances.
We define the Hamming distance $d_h(\mathbf{x_1}, \mathbf{x_2})$ between two vectors $\mathbf{x_1}, \mathbf{x_2}$as the number

	Yes	No
Flatscreen TV	348 (82%)	77 (18%)
Traditional TV	244 (57%)	181 (43%)
Microwave	184 (43%)	241 (57%)
Computer	342 (80%)	83 (20%)
Washing Machine	419 (99%)	6 (1%)
Tumble dryer	45 (11%)	380 (89%)
Fridge	420 (99%)	5 (1%)
Freezer	275 (65%)	150 (35%)
Vacuum Cleaner	347 (82%)	78 (18%)
Dishwasher	255 (60%)	170 (40%)
Hairdryer	409 (96%)	16 (4%)
Iron	417 (98%)	8 (2%)
Electric Oven	390 (92%)	35 (8%)
Electric Stove	83 (20%)	342 (80%)
Air Conditioning	55 (13%)	370 (87%)
Portable Air Conditioning	25 (6%)	400 (94%)
Cooking Robot	161 (38%)	264 (62%)
Polisher	28 (7%)	397 (93%)
Hi-Fi System	233 (55%)	192 (45%)
Electric Water Heater	63 (15%)	362 (85%)
Electric Air Heater	101 (24%)	324 (76%)
Gaming Consoles	140 (33%)	285 (67%)
DVD Player	275 (65%)	150 (35%)
PC Printer	256 (60%)	169 (40%)
Hydraulic Pump	40 (9%)	385 (91%)
Internet-Connected Smartphone	228 (54%)	197 (46%)
Tablet Device	117 (28%)	308 (72%)
MP3 Player	147 (35%)	278 (65%)
Portable Gaming Console	104 (24%)	321 (76%)
Digital Camera	264 (62%)	161 (38%)
Digital Videocamera	169 (40%)	256 (60%)
Radiators	320 (75%)	105 (25%)
Wood/Pellet Stove	114 (27%)	311 (73%)
Wood Stove with Ducted Air	72 (17%)	353 (83%)

Table B: List of Appliances with contingency table and ownership percentages

of digits in which they differ, e.g

$$\begin{aligned} d_h(00111, 11000) &= 5 \\ d_h(10, 11) &= 1 \\ d_h(101, 110) &= 2 \end{aligned}$$

It can be easily proven that the Hamming distance satisfies the usual conditions required to a metric, i.e.

- $d_h(\mathbf{x_1}, \mathbf{x_2}) \geq 0$, and $d_h(\mathbf{x_1}, \mathbf{x_2}) = 0 \longleftrightarrow \mathbf{x_1} = \mathbf{x_2}$
- $d_h(\mathbf{x_1}, \mathbf{x_2}) = d_h(\mathbf{x_2}, \mathbf{x_1})$
- $d_h(\mathbf{x_1}, \mathbf{x_3}) \leq d_h(\mathbf{x_1}, \mathbf{x_2}) + d_h(\mathbf{x_2}, \mathbf{x_3})$

A graphical representation of the distance matrix calculated over the columns of our dataset can be found in Figure A. The idea of clustering columns will provide us with groups of appliances that tend to appear together within an household.

Hierarchical Clustering

After having identified a proper dissimilarity measure between data objects, we can proceed to develop a clustering.

The idea of Hierarchical Clustering methods is to develop a Hierarchical representation of the data objects, in which the groups at each level of the hiearchy are created by merging clusters at the next lower level. The lowest level is composed by clusters made by only one observation, while the top one is made by one single cluster composed by the whole dataset.
While some methods working with a top-down approach can be found [15], the most common choice is the so called agglomerative clustering, that uses a bottom-up method. Agglomerative strategies start at the bottom, with completely separated data objects, and at each level recursively merge a selected pair of clusters into a single one. This produces a grouping at the next higher level with one less cluster. The criterion used to choose the pair to be merged is to select the two groups with the smallest inter-group dissimilarity.

Recursive binary agglomeration can be represented as a rooted binary tree, where the nodes represent groups. The root node represents the entire set of data objects, while the N terminal leaves are the so called singleton clusters.
It can be easily proven that all agglomerative clustering methods possess a monotonicity property, that is the dissimiliarity between merged clusters is monotonically increasing with the level of the merger. Thus the binary tree can be plotted so that the height of the node is proportional to the value of the intergroup similarity

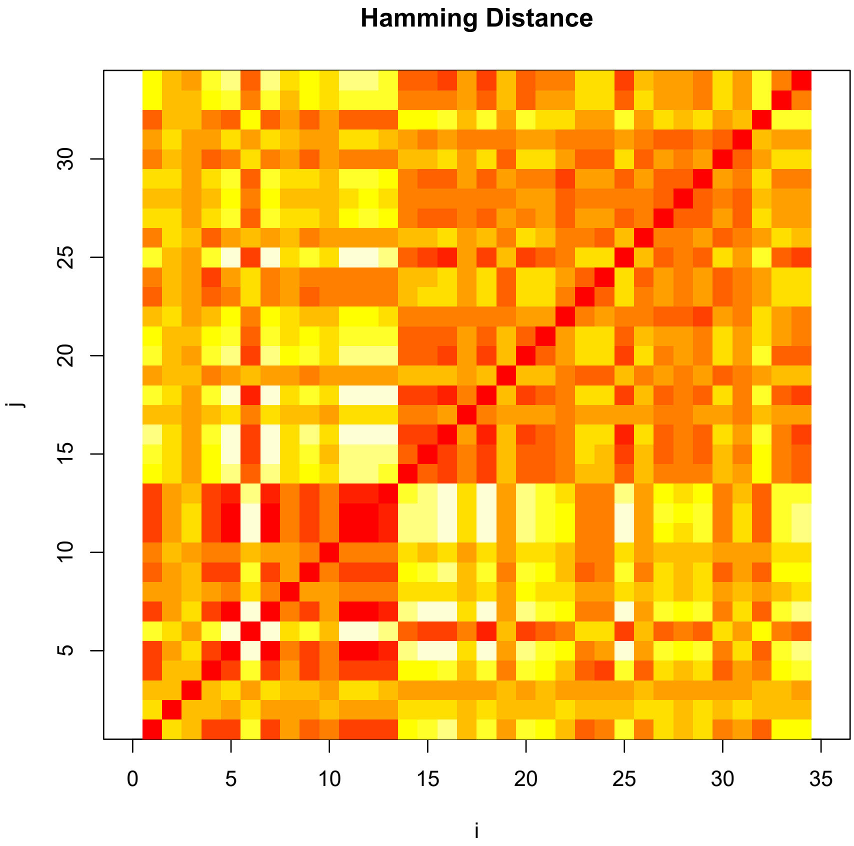

Figure A: Graphical representation of the Hamming distance matrix

between its two daughters, and the terminal nodes (the single observations) are all plotted at zero height.
This type of graphical representation is called a *dendrogram.*
A dendrogram provides a highly interpretable complete description of the hierarchical clustering in a graphical format.

The extent to which the hierarchical structure produced by a dendrogram represents the dataset itself can be measured by using the so called *cophenetic correlation coefficient,* that is the correlation between the $N(N-1)/2$ pairwise observation dissimilarities $d_{i,j}$ and their corresponding *cophenetic* dissimilarities $c_{i,j}$, that is defined as the amount of intergroup dissimilarity at which data objects i and j are firstly merged together in the same cluster.

We now briefly describe how the agglomerative clustering algorithms work
The starting point is the situation where every observation represents a singleton cluster. At each of the $N-1$steps, the least dissimilar clusters are merged into a single one, producing one less cluster at the next higher level.
While the dissimilarity between data objects has already been identified, we now must define a measure of dissimilarity between clusters.

Let G and H be two clusters, for which we want to define a dissimilarity measure $d(G, H)$. This measure is clearly computed from the set of pairwise object dissimilarities $d_{i,j}$,where $i \in G$ and $j \in H$.

- *Single Linkage* (SL), or nearest neighbour technique takes the inter-group dissimiliarity to be the one of the least dissimilar pair

$$d_{SL}(G,H) = \min_{i \in G, j \in H} d_{i,j} \tag{17}$$

- *Complete Linkage* (CL) or furthest neighbour takes the maximum inter-group dissimilarity instead

$$d_{CL}(G,H) = \max_{i \in G, j \in H} d_{i,j} \tag{18}$$

- *Average Linkage* (AL) uses the average dissimilarity between the groups

$$d_{AL}(G,H) = \frac{1}{N_G N_H} \sum_{i \in G} \sum_{j \in H} d_{i,j} \tag{19}$$

- *Ward Linkage* (WL) considers the sum of squared distance between points

$$d_{WL}(G,H) = \sum_{i \in G} \sum_{j \in H} d^2_{i,j} \tag{20}$$

To assess the robustness of the clustering procedure, three other linkage techniques were tested. The dendrograms generated by using Complete, Single, Average and

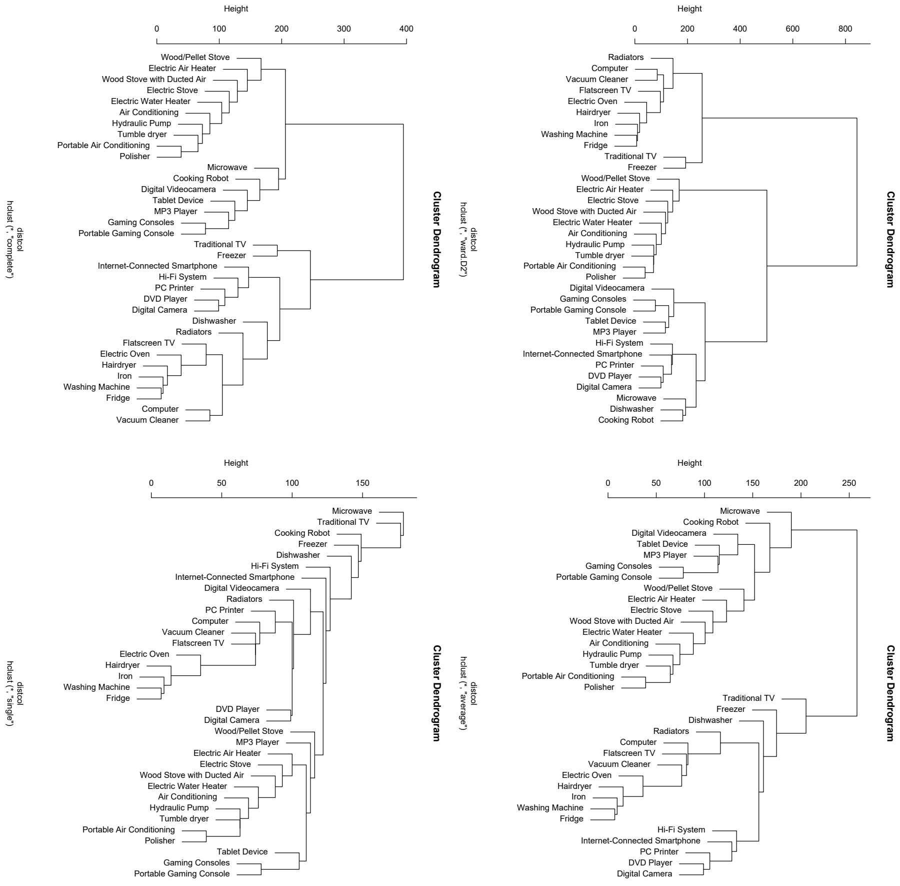

Figure B: Dendrograms generated using different linkage methods.

Ward linkage can be seen in Figure A, while the Cophenetic Coefficient (CC) values associated to the four different linkage techniques can be seen in Table 3.We can see that while complete and average linkage provide very high CCs, the Ward [?] linkage gives a very powerful and insightful explanation of data, and retains a very good CC of 0.712, that yields a sufficiently high CC, with a very powerful explanation given by this particular clustering method.

A color-coded dendrogram, where Basic Appliances are shown in blue, low tech in yellow and high tech in red can be seen in Figure B

Linkage Method	Cophenetic Correlation Coefficient
Single Linkage	0.426
Complete Linkage	0.815
Average Linkage	0.827
Ward Linkage	0.712

Table C: Cophenetic coefficients of the various linkage techniques used

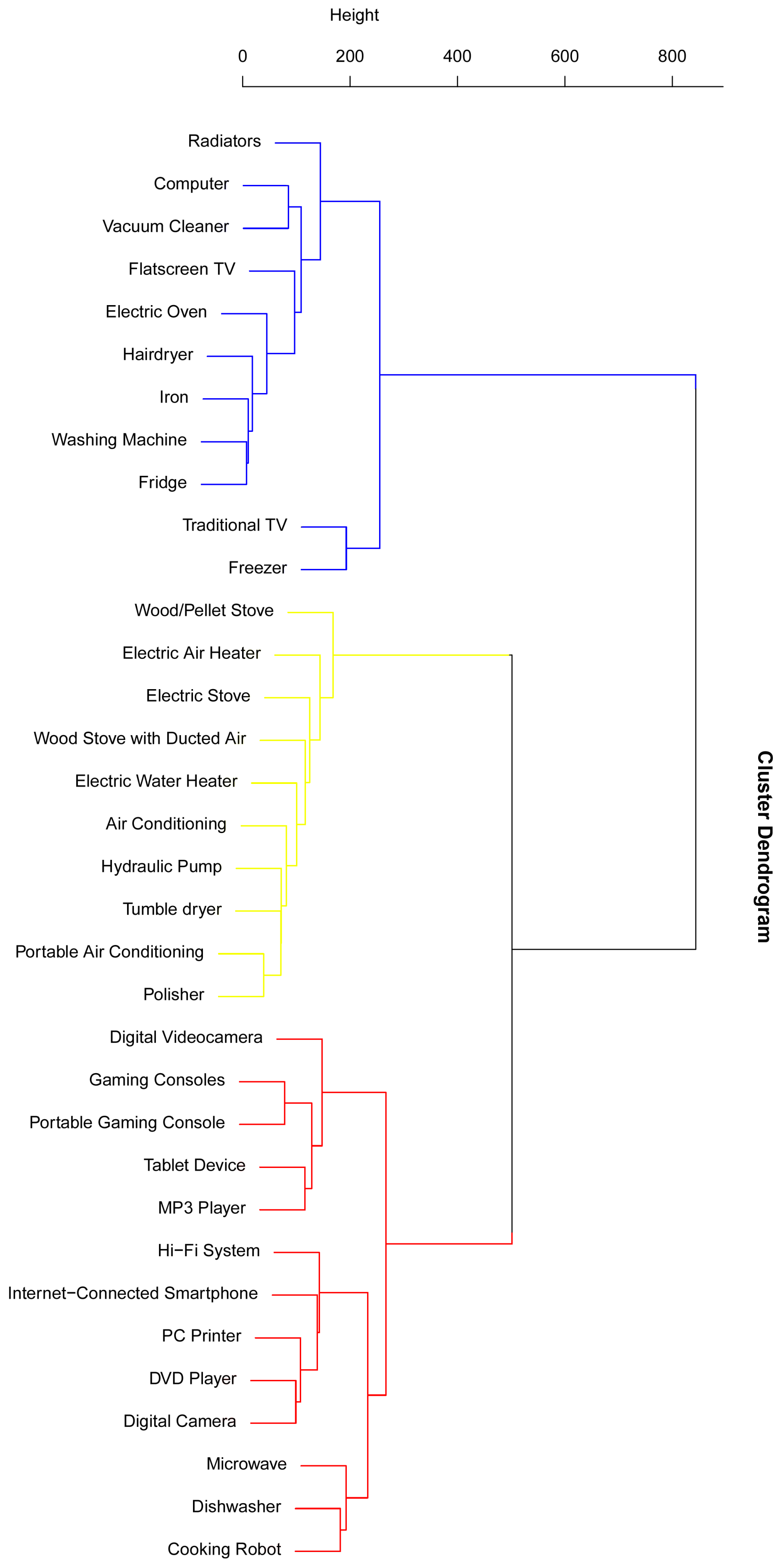

Figure C: Ward linkage dendrogram with highlighted clusters

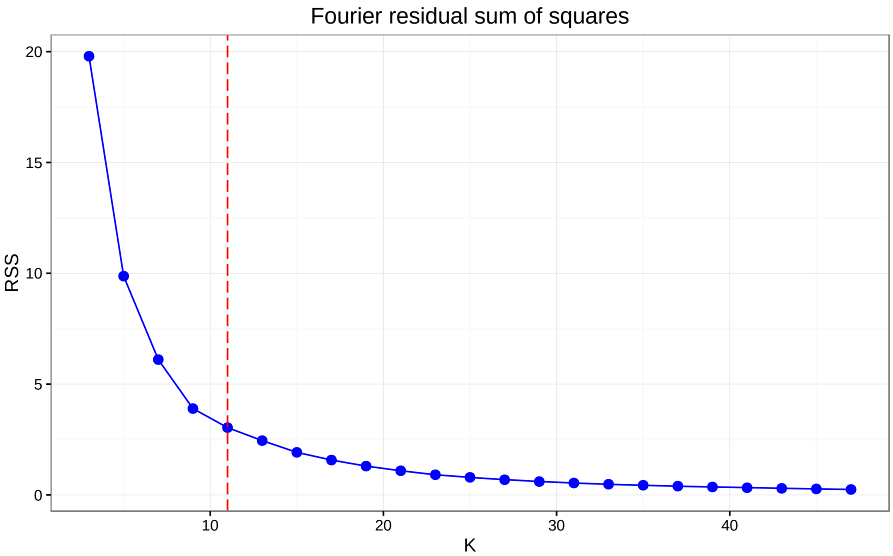

Figure D: Residual sum of squares of Fourier expansion

Functional Smoothing

We present in Figure C the Residual Sum of Squares obtained projecting our data over Fourier bases with increasing size. We have a clear elbow (and so, a change in the percentage of variance explained by an increment of the basis size) for $K = 11$.

0.7 Model Selection

A reasonable assumption about the energy disaggregation problem is that a part of energy consumption is uncorrelated with appliance ownership (e.g. electric lights, energy dispersions), and for this reason we will include a functional intercept in our regression model. To test this assumption, and to perform some model selection, we start by estimating the following functional on scalar regression model

$$y(t)_f = \beta_{intercept}(t) + \beta_{Basic}(t)I_{fB} + \beta_{Hi.tech}(t)I_{fH} + \beta_{Lo.tech}(t)I_{fL} + \epsilon(t) \qquad (21)$$

Where $t \in (0, 24)$, $I_{f,B}$, $I_{f,H}$, $I_{f,L}$are respectively the values of penetration of basic, hi-tech and low tech appliances. and $y(t)_f$ is the average energy consumption function over time per household and $\epsilon(t)$ is a zero-mean unpredictable error component, with covariance structure $\Sigma(t, t')$. We recall that in this case$n$, the number of statistical units, is 425.

The regression results can be seen in Figure D. We can see that $\beta_{intercept}(t)$ is indeed significant over the whole domain, meaning that our assumption is correct. Moreover $\beta_{Basic}(t)$ is never significant over the domain, probably due to collinearity

issues.. If we drop the Basic appliances ownership index, we can see our regression model as the sum of a consumption baseline, plus variable terms bound to High tech and Lo tech appliances ownership indices, thus getting the following functional on scalar regression model

$$y(t)_f = \beta_{Baseline}(t) + \beta_{Hi.tech}(t)I_{fH} + \beta_{Lo.tech}(t)I_{fL} + \epsilon(t) \tag{22}$$

0.8 Models Robustness to Smoothing and Specification

To investigate the robustness of the inferential results with respect to the choice of the Fourier basis used for the data smoothing procedure, we have performed a test varying this parameter, run the model in Equation 22 on data smoothed halving and doubling the size of the Fourier basis. In particular, starting from the original analysis based on a basis expansion of size $K = 11$, we explore an under-smoothed case with $K = 7$ and an over-smoothed case with $K = 23$.

The F-Test and t-Tests for the two explored cases are reported respectively in Figure F and Figure G. As it was the case with the previous analysis, the grey shading in the plots signals the significance of the test at a 5% level. We can see how the results of the regression analysis suffer very minor changes in the shapes of the functional regression coefficients, the areas of the domain where the tests are significant are essentially unchanged (with the exception of night time for the high-tech appliances group, but with very low values of the coefficient) and, essentially, that our interpretation of the results remains substantially unchanged. This result is consistent with [20], where it is shown that IWT is particularly robust with respect to smoothing.

To test the robustness with respect to the specification of our disaggregation model, we vary the specification of the functional regression equation, by adding additional household-specific covariates. in particular, we specify a model of the form:

$$y(t)_f = \beta_{Baseline}(t)+\beta_{Hi.tech}(t)I_{fH}+\beta_{Lo.tech}(t)I_{fL}+\beta_{nr.people}(t)x_{f1}+\beta_{typeind}(t)x_{f2} + \beta_{rooms}(t)x_{f3} + \epsilon(t)_f \tag{23}$$

Where$I_{f,H}$, $I_{f,L}$ are the values of ownership of hi-tech and low tech appliances respectively, $x_1$is the number of people living in a given household, $x_{f2}$is a dummy variable with value 0 if the house is an apartment, and 1 otherwise, and $x_{f3}$is the number of rooms a house is composed of, $\beta(t)_x \forall x\ in$ \{Baseline, Hi.Tech, Lo.Tech, nr.people, typeind, rooms} are functional regression coefficients, $y(t)_f$ is the average

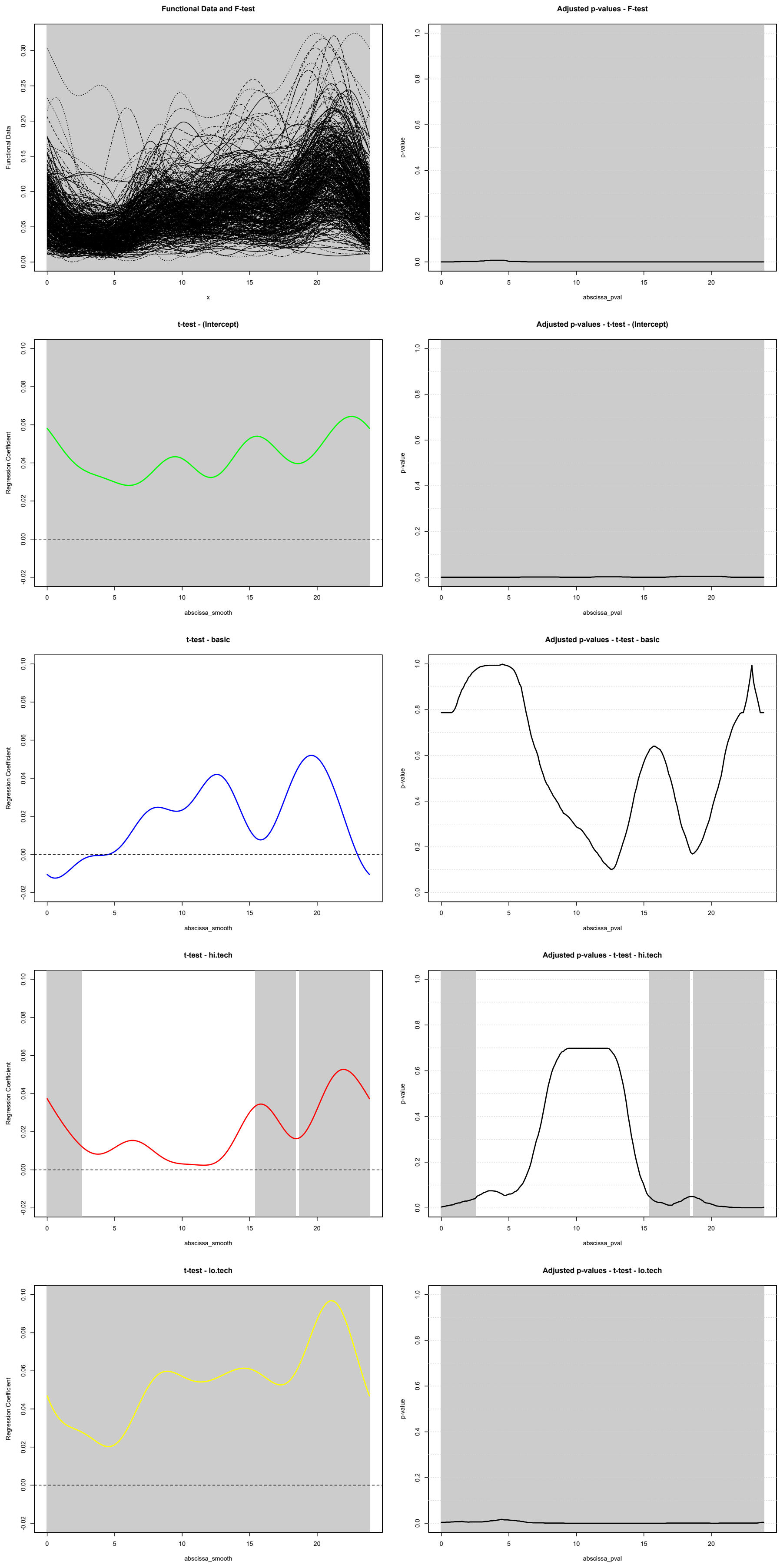

Figure E: F-Tests and t-Tests for Regression model in Equation 21

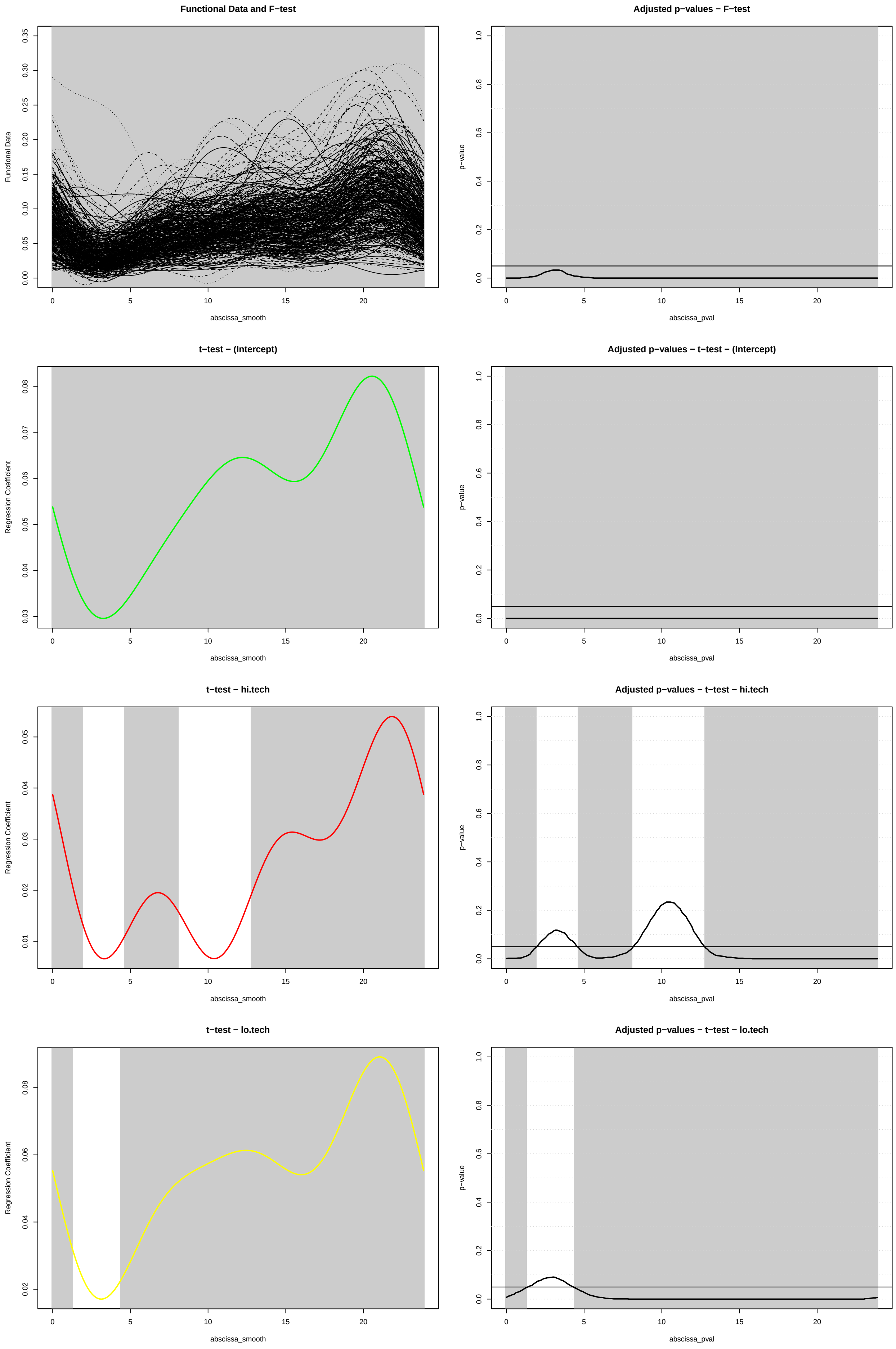

Figure F: F-Test and t-Tests for regression model in Equation 22, $K = 7$

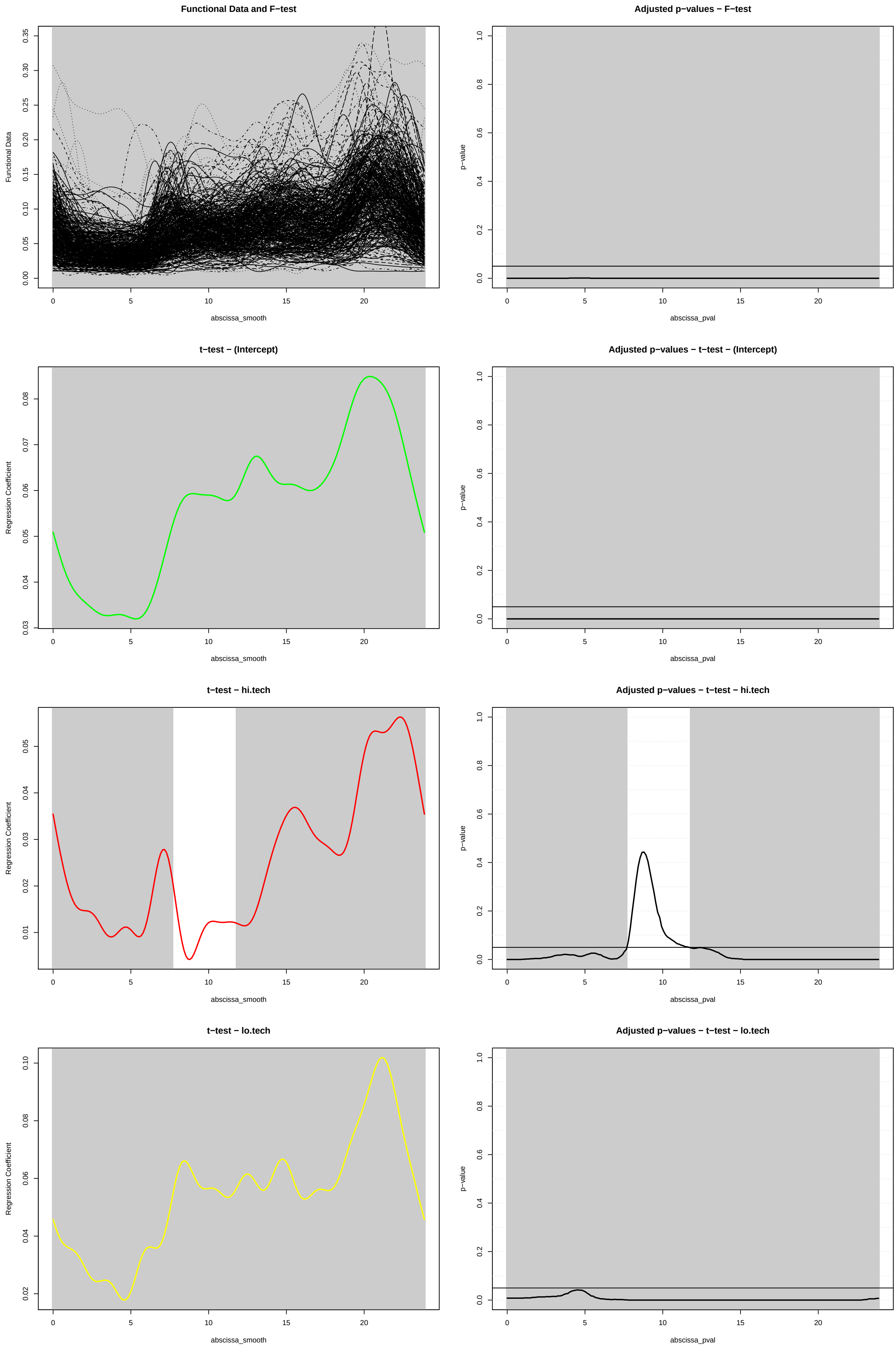

Figure G: F-Test and t-Tests for regression model in Equation 22, $K = 23$

energy consumption function over time per household f and $\epsilon(t)_f$ is a zero-mean unpredictable error component, with covariance structure $\Sigma(t,t')$. The results of the tests for the model in equation 23 can be found in Figure E. We observe that the portions of domain that are significant for hi tech and low tech coefficients are somewhat smaller than in the original regressions, but the magnitude and shape of the coefficients remains the same. The baseline component, instead, loses significance. This fact has a very clear explanation: in the previous specification the $\beta_{Baseline}(t)$ component was modelling that part of energy consumption that wasn't explained by the appliance ownership patterns (among all, electric lighting and energy dispersions), while household specific covariates such as the number of people, or the type of house are able to split the very aggregated baseline in more specific and refined components.

We are able to test the robustness also on the speed of change in consumption during the day. The specified model is the following:

$$Dy(t)_f = \beta_{Baseline}(t) + \beta_{Hi.tech}(t)I_{fH} + \beta_{Lo.tech}(t)I_{fL} + \beta_{nr.people}(t)x_{f1} + \beta_{typeind}(t)x_{f2} + \beta_{rooms}(t)x_{f3} + \epsilon(t)_f \quad (24)$$

Where$I_{f,H}$, $I_{f,L}$ are the values of ownership of hi-tech and low tech appliances respectively, $x_1$is the number of people living in a given household, $x_{f2}$is a dummy variable with value 0 if the house is an apartment, and 1 otherwise, and $x_{f3}$is the number of rooms a house is composed of, $\beta(t)_x \forall x\ in\ \{Baseline,\ Hi.Tech,\ Lo.Tech,\ nr.people,\ typeind,\ rooms\}$ are functional regression coefficients, $Dy(t)_f$ is the first derivative of the average energy consumption over time per household f and $\epsilon(t)_f$ is a zero-mean unpredictable error component, with covariance structure $\Sigma(t,t')$. The results of the tests for the model in equation 24 can be found in Figure F. In this case, too, some significance of the role of appliances is lost to the additional covariates: in particular a reduced significant domain for the appliance-related coefficients, together with the lack of significance for the Baseline component. The explanation is the same as before: the baseline coefficient was modelling the part of electricity consumption, that was not explained by ownership patterns. This component can clearly related to household specific covariates, such as the number of people present in the household, that generally increases electricity consumption during the day, with a pattern that resembles the average load curve. The type of house is significant too: independent houses have an average load curve that is shifted upwards compared to the other households.

We've also performed a robustness test on the diff-in-diff regression to assess the impact of the introduction of real-time feedback. The specified model is:

$$
\begin{aligned}
log\Delta(t)_f &= \beta_{Intercept}(t) + \beta_{Hi.tech}(t)\log(I_{f,H}) + \beta_{Lo.tech}\log(I_{f,L}) \\
&+ \beta_{nr.people}(t)log(x_{f1}) + \beta_{typeind}(t)x_{f2} + \beta_{rooms}(t)log(x_{f3}) + \epsilon(t) \quad (25)
\end{aligned}
$$

The results of the tests ofr the model in equation 25 can be found in Figure G. Adding covariates renders the intercept term non significant. This means that there is correlation between regressors, and that indeed some part of the energy savings after the introduction of real-time feedback can be attributed to household specific covariates. However, the signal is too weak to appear significant.

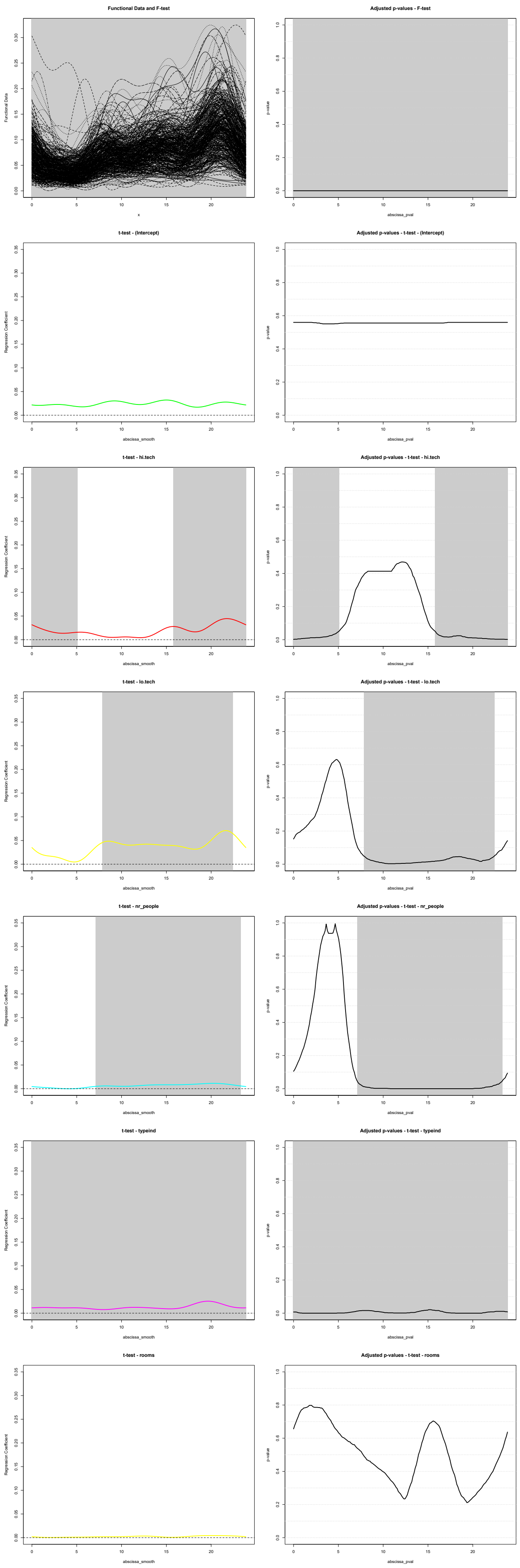

Figure H: F-Test and t-Tests for regression model 23

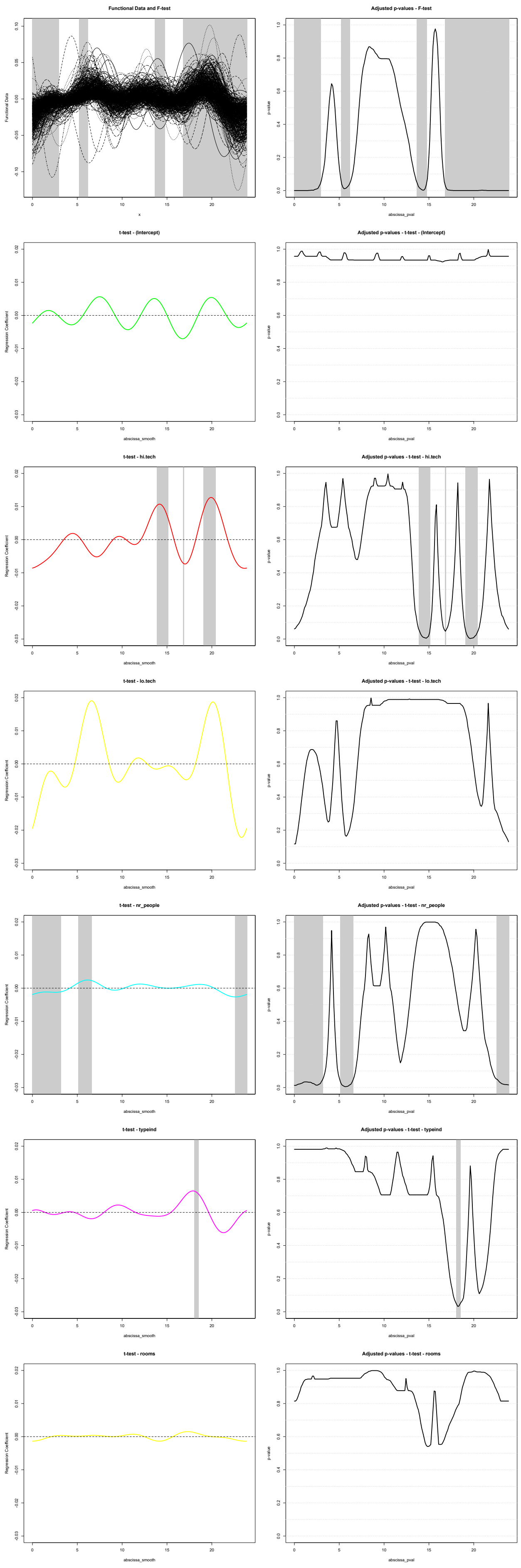

Figure I: F-Test and t-Tests for regression model 24

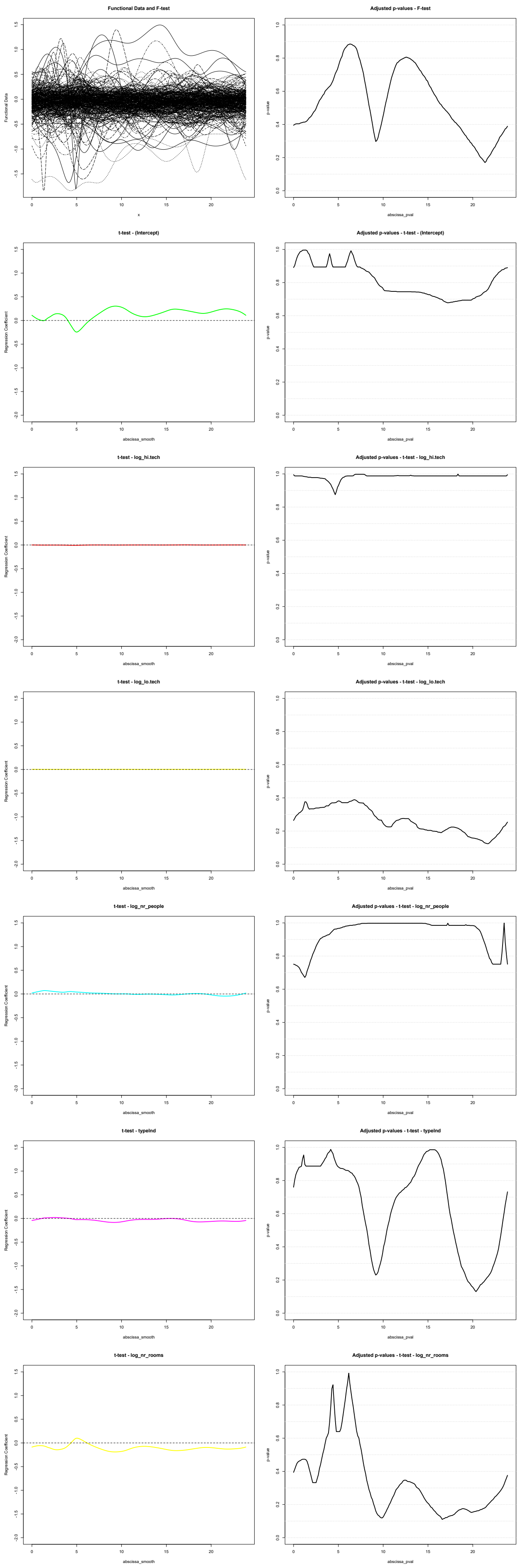

Figure J: F-Test and t-Tests for regression model 25

Bibliography

[1] Marti J. Anderson and Pierre Legendre. An empirical comparison of permutation methods for tests of partial regression coefficients in a linear model. *Journal of Statistical Computation and Simulation*, 62(3):271–303, 1999.

[2] Livio Corain, Viatcheslav B. Melas, Andrey Pepelyshev, and Luigi Salmaso. New insights on permutation approach for hypothesis testing on functional data. *Advances in Data Analysis and Classification*, 8(3):339–356, 2014.

[3] Antonio Cuevas. A partial overview of the theory of statistics with functional data. *Journal of Statistical Planning and Inference*, 147:1–23, 2014.

[4] Frederic Ferraty. *The Oxford handbook of functional data analysis.* Oxford University Press, 2011.

[5] Frederic Ferraty and Philippe Vieu. *Nonparametric Functional Data Analysis - Theory and Practice.* Springer, 2006.

[6] David Freedman and David Lane. A Nonstochastic Interpretation of Reported Significance Levels. *Journal of Business & Economic Statistics*, 1(4):292–298, 1983.

[7] Ulf Grenander. Stochastic processes and statistical inference. *Arkiv For Matematik*, 1(17):195–276, 1950.

[8] Peter Hall and Ingrid Van Keilegom. Two-Sample Tests in Functional Data Analysis Starting From Discrete Data. *Statistica Sinica*, 17:1511–1531, 2007.

[9] Peter Hall and Nader Tajvidi. Permutation tests for equality of distributions in high-dimensional settings. *Biometrika*, 89(2):359–374, 2002.

[10] Richard W Hamming. Error detecting and error correcting codes. *Bell System Technical Journal*, 29(2):147–160, 1950.

[11] Trevor Hastie, Robert Tibshirani, and Jerome Friedman. *The Elements of Statistical Learning: Data Mining, Inference, and Prediction.* Springer, 2009.

[12] Lajos Horváth and Piotr Kokoszka. *Inference for Functional Data with Applications.* Springer, 2012.

[13] Tailen Hsing and Randall Eubank. *Theoretical Foundations of Functional Data Analysis, with an Introduction to Linear Operators.* Wiley, 2015.

[14] I.T. Jolliffe. *Principal component analysis.* Springer, 2002.

[15] Leonard Kaufman and Peter J Rousseeuw. *Finding Groups in Data: An Introduction to Cluster Analysis.* Wiley, 2005.

[16] T L Lai, Herbert Robbins, and C Z Wei. Strong consistency of least squares estimates in multiple regression. *Journal of Multivariate Analysis*, 9(3):343–361, 1979.

[17] Fortunato Pesarin and Luigi Salmaso. *Permutation Tests for Complex Data.* Wiley, 2010.

[18] A. Pini and S. Vantini. Interval-wise testing for functional data. *Journal of Nonparametric Statistics*, 29(2):407–424, 2017.

[19] Alessia Pini and Simone Vantini. The interval testing procedure: A general framework for inference in functional data analysis. *Biometrics*, 72(3):835–845, 2016.

[20] Alessia Pini, Simone Vantini, Bianca Maria Colosimo, and Marco Grasso. Domain-selective functional analysis of variance for supervised statistical profile monitoring of signal data. *Journal of the Royal Statistical Society: Series C (Applied Statistics)*, 67(1):55–81, January 2018.

[21] J. O. Ramsay. When the data are functions. *Psychometrika*, 47(4):379–396, 1982.

[22] J. O. Ramsay and B. W. Silverman, editors. *Applied Functional Data Analysis: Methods and Case Studies.* Springer, 2002.

[23] J.O. Ramsay and C.J. Dalzell. Some Tools for Functional Data Analysis. *Journal of the Royal Statistical Society. Series B*, 53(3):539–572, 1991.

[24] J.O. Ramsay and B. W. Silverman. *Functional Data Analysis.* Springer, 2005.

[25] C. Radhakrishna Rao. Some Statistical Methods for Comparison of Growth Curves. *Biometrics*, 14(1):1–17, 1958.

[26] Dan Shen, Haipeng Shen, Shankar Bhamidi, Yolanda Muñoz Maldonado, Yongdai Kim, and J S Marron. Functional Data Analysis of Tree Data Objects. *Journal of computational and graphical statistics*, 23(2):418–438, 2014.

[27] Thaddeus Tarpey and Kimberly K. J. Kinateder. Clustering functional data. *Journal of Classification*, 20(1):93–114, 2003.

[28] Olga Vsevolozhskaya, Mark Greenwood, and Dmitri Holodov. Pairwise comparison of treatment levels in functional analysis of variance with application to erythrocyte hemolysis. *Annals of Applied Statistics*, 8(2):905–925, 2014.

[29] Jane-Ling Wang, Jeng-Min Chiou, and Hans-Georg Mueller. Review of Functional Data Analysis. *http://arxiv.org/abs/1507.05135*, 2015.

[30] Anderson M. Winkler, Gerard R. Ridgway, Matthew A. Webster, Stephen M. Smith, and Thomas E. Nichols. Permutation inference for the general linear model. *Neuroimage*, 92:381–397, 2014.

[31] Jin-Ting Zhang. *Analysis of variance for functional data.* CRC Press, 2014.
